# Pyroxd1 is essential for murine viability with the homozygous N155S recurrent variant linked to myopathy, muscle hypotrophy and osteopenia

**DOI:** 10.1186/s40478-026-02259-1

**Published:** 2026-03-02

**Authors:** Frances J. Evesson, Gregory Dziaduch, Joe Yasa, Heather A. Best, Leonit Kiriaev, Ignatius Pang, Vanessa Jones, Emma Kettle, Katharine Zhang, Ann-Katrin Piper, Jesse R. Wark, Isaac Scott, Himanshu Joshi, R. Bryan Sutton, Patrick P. L. Tam, Peter J. Houweling, Mark E. Graham, Michaela Yuen, Frances A. Lemcket, Sandra T. Cooper

**Affiliations:** 1 School of Medical Sciences, Faculty of Medicine and Health, The University of Sydney, Sydney, Australia; 2 Kids Neuroscience Centre, The Children’s Hospital at Westmead, Sydney, Australia; 3 Children’s Medical Research Institute, Westmead, Sydney, Australia; 4 Present Address: Current position: Garvan Institute of Medical Research, Sydney, Australia; 5 Sydney Medical School, Faculty of Medicine and Health, The University of Sydney, Sydney, Australia; 6 Present Address: Current position: Environmental Scientist, Morphum Environmental Limited, Auckland, New Zealand; 7 Muscle Research, Murdoch Children’s Research Institute, Melbourne, Australia; 8 Westmead Research Hub Electron Microscope Core Facility, The Westmead Institute for Medical Research, Sydney, Australia; 9 Present Address: Current position: DeepTech Incubator, Macquarie University, Sydney, Australia; 10 Present Address: Current position: Molecular Horizons and School of Science, University of Wollongong, Wollongong, Australia; 11 Texas Tech University Health Sciences Center, Lubbock, TX USA

## Abstract

Biallelic variants in *PYROXD1* are associated with a life limiting muscle and connective tissue disorder characterised by generalised muscle weakness, breathing and feeding difficulties, distal laxity, hypernasal speech, blue sclera and osteopenia. *PYROXD1* encodes an oxidoreductase implicated in mitochondrial function and tRNA ligase activity. Herein we present the first comprehensive suite of mouse models designed to elucidate PYROXD1 redox functions and the underlying pathogenetic basis for *PYROXD1* disorders. A LacZ reporter strain reveals ubiquitous expression of *Pyroxd1*, particularly in the developing head, eye, heart and skin. Complete knockout (KO) of *Pyroxd1* resulted in embryonic lethality between ED4.5–E9.5, suggesting Pyroxd1 performs a unique function during embryogenesis that cannot be substituted by other redox enzymes. Skeletal muscle tissue-specific KO, or stage-specific tamoxifen-induced KO during gestation or post-weaning, were viable but not sustainable models. Tamoxifen-inducible KO cell lines derived from these models provide valuable tools for dissecting Pyroxd1 function. Homozygous mice harbouring the recurrent human variant NM_024854.5:c.464A > G;p.(N155S), termed *Pyroxd1*_N155S_, phenocopied a severe PYROXD1 disorder, presenting from ~ 10 weeks of age with a progressive myopathy, myofibrillar disorganisation, decreased contractile strength, muscle hypotrophy and osteopenia. Conversely, homozygous *Pyroxd1*_N155G_ mice, created incidentally during CRISPR editing, were phenotypically normal and provide an important benign control. Proteomic analyses reveal distinct molecular signatures between acute *Pyroxd1* KO and *Pyroxd1*_N155S_ models_,_ highlighting differential effects of complete loss-of-function (KO) compared to partial enzymatic activity (N155S). Pyroxd1 activity is critical for numerous cell essential processes, including protein biosynthesis and turnover, tRNA ligase complex activity and the unfolded protein response, mitochondrial respiratory chain function (especially complex 1), with an implicated broader role in the exon-ligation step of pre-mRNA splicing. Together, these murine *Pyroxd1* models establish an enabling platform for gaining mechanistic insight and guiding the path of future therapeutic development for PYROXD1 disorders.

**Supplementary Information:**

The online version contains supplementary material available at 10.1186/s40478-026-02259-1.

## Introduction

PYROXD1 (Pyridine Nucleotide-Disulfide Oxidoreductase Domain-Containing Protein 1, PYROXD1 in humans, Pyroxd1 in mice) is an oxidoreductase vital for the maintenance of normal cellular redox balance [1, 2]. Biallelic variants in *PYROXD1* cause a muscle and connective tissue disorder characterised by muscle weakness, respiratory and feeding difficulties, distal joint laxity and osteopenia [2–9]. There is currently no available therapy for *PYROXD1* disorders, and the condition is life-limiting.

*PYROXD1* disorders are characterised histologically by skeletal muscle fibre size variability, internalised nuclei, core-like regions devoid of mitochondrial activity, protein accumulations and electron dense nemaline rod inclusions [3]. Additionally, we recently identified significant connective tissue features in two affected families [7] and we and others have reported PYROXD1 patients with decrement on repetitive nerve stimulation [7, 10]. As an ultra-rare condition with currently fewer than 50 patients known to be diagnosed worldwide, the true prevalence and phenotypic spectrum of *PYROXD1* related conditions remains unclear. The recurrent human variant, Chr12(GRCh38):g.21452130A > G; NM_024854.5:c.464A > G;p.(N155S) has been identified on at least one allele in 80% of patients diagnosed to date [7], associated with both early- [3, 7] and adult-onset [2, 4–6, 8, 9] presentations. Individuals with homozygous missense variants (including p.N155S) have a milder disease presentation with later disease onset, compared to patients with compound heterozygous PYROXD1 variants, such as p.N155S *in trans* with a loss-of-function variant [7].

PYROXD1 is essential for cell viability [11–13]. It has a nuclear-cytoplasmic localisation [3, 5] and previous research has identified a role for PYROXD1 in mitochondrial function [2] and regulation of the tRNA ligase complex [1, 14]. While these roles reflect important aspects of PYROXD1 function, neither alone can fully explain the pathology of PYROXD1 disease. To better elucidate the redox role(s) of PYROXD1 at both cellular and organism levels, we generated a suite of mouse and cell models harbouring a *Pyroxd1-*lacZ reporter, global *Pyroxd1* KO, inducible *Pyroxd1* KO, skeletal muscle *Pyroxd1* KO and homozygous *Pyroxd1*_N155S_ missense alleles. Using these models, we demonstrate a critical role for PYROXD1 in mitochondrial function and provide evidence for novel roles for PYROXD1 in RNA processing and protein synthesis. Our work now extends the knowledge base of PYROXD1 disorders and delivers vital models for the field.

## Results

### Pyroxd1 is expressed throughout mouse development, and in all mouse tissues

**Fig. 1:**
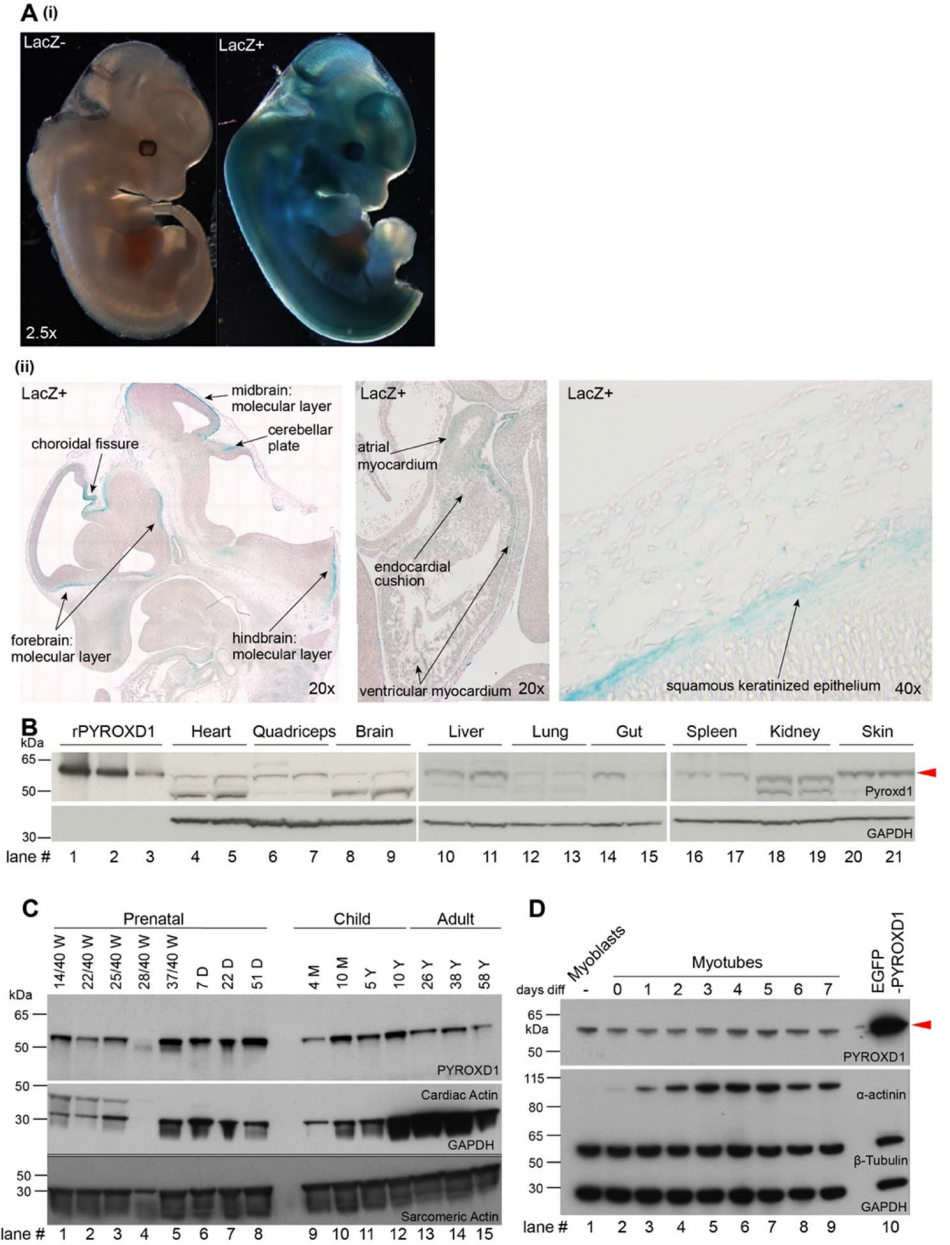
PYROXD1 is expressed throughout murine and human development. **A** *Pyroxd1-lacZ* expression in (i) ED12.5 *Pyroxd1*^Tm1a/WT^ embryo and (ii) in tissues of the brain, heart and skin of ED15.5 embryo. **B** Western blot shows PYROXD1 expression in adult mouse tissues (lanes 4–21). PYROXD1 specific band (red arrowhead) with three decreasing concentrations of purified recombinant PYROXD1 protein (rPYROXD1, lanes 1–3) loaded to demonstrate that detection is in the linear range for the antibody. (n = 4 mice, shown for n = 2). **C**, **D** Western blot showing PYROXD1 expression in **C** human skeletal muscle at ages from gestation to adulthood and **D** in cultured proliferating myoblasts (Lane 1) to myotubes (Lanes 2–9, Days 0–7 in vitro differentiation). Lysate from cells overexpressing EGFP-PYROXD1 is included as a positive control (lane 10)

*Pyroxd1* expression was examined in *Pyroxd1 Tm1a* mouse (*Pyroxd1*^Tm1a/WT^) embryos containing a gene-trap *lacZ* cassette encoding β-galactosidase controlled by the *Pyroxd1* promoter (Supplementary Fig. 1A). Widespread β-galactosidase expression, reflecting *Pyroxd1* activity, was observed in embryonic day (ED) 12.5 embryos (Fig. 1Ai), and in the brain, heart and skin of ED 15.5 embryos (Fig. 1Aii). In wild type adult mice, PYROXD1 protein expression was detected in a wide range of tissues including the heart, skeletal muscle, brain, liver, spleen, kidney and skin (Fig. 1B). Given the predominant skeletal muscle phenotype of PYROXD1 disorders, we profiled PYROXD1 expression in human muscle and detected PYROXD1 expression at all ages tested for which samples were available, from prenatal stages to 58 years age (Fig. 1C), and in cultured human primary myoblasts and myotubes at 0 to 8 days of in vitro differentiation (Fig. 1D).

### Embryonic lethality of Pyroxd1 knockout mice between ED4.5 and ED9.5

While the heterozygous *Pyroxd1*^Tm1a/WT^ mice were viable, homozygous *Pyroxd1*^Tm1a/Tm1a^ mice, which harbour the loss of function Tm1a mutation of *Pyroxd1*, were absent among 165 offspring from *Pyroxd1*^Tm1a/WT^ x *Pyroxd1*^Tm1a/WT^crosses. Incidentally, three other single-gene knock out studies also demonstrated that homozygous LOF mutation was incompatible with cellular viability [11–13]. To pinpoint the timing of embryonic lethality, we examined embryos from crosses of heterozygous *Pyroxd1*^Tm1a/WT^ mice. In vitro culture of zygotes collected at ED0.5 from *Pyroxd1*^Tm1a/WT^ females mice crossed with *Pyroxd1*^Tm1a/WT^ males produced 2 homozygous *Pyroxd1*^Tm1a/Tm1a^ blastocysts that were phenotypically indistinguishable from 3 heterozygous blastocysts and 3 wild type blastocysts. However, at ED10, no viable homozygous embryos were found among 17 embryos collected from two litters. Our data therefore suggests PYROXD1 protein sourced from the gametes [15] can sustain the viability of homozygous *Pyroxd1* embryo till at least the blastocyst stage, and that the homozygous embryos were lost between ED4.5 and ED9.5.

### MyoD-Cre driven Pyroxd1 KO results in widespread Pyroxd1 KO and is lethal for neonatal mice

Given the primary skeletal muscle pathology of *PYROXD1* myopathy and lethality of global *Pyroxd1* KO mice, we created a skeletal-muscle-specific *Pyroxd1* knockout KO mouse model. This required two breeding steps: (1) Flippase recombination to remove the LacZ reporter cassette to generate mice with floxed *Pyroxd1* alleles (PFLP^+/+^) (Supplementary Fig. 1B*i*); (2) Crossing PFLP^+/+^ mice with mice carrying the MyoD^Cre^ transgene to achieve muscle-specific knock out of *Pyroxd1* (Supplementary Fig. 1B*ii*). These mice survived until birth with expected genotype ratios, though died shortly after birth (postnatal day 2, n = 2/13 pups). Interpretation of the probable cause of death was complicated by detectable Cre mediated recombination of the floxed allele in tissues others than skeletal muscle (e.g. in gDNA from ear and tail clips, not shown but previously described for other mouse lines [16]), indicating Pyroxd1 KO extended to non-skeletal muscle tissues perhaps contributing to neonatal death. For ethical reasons, this breeding strategy was not pursued.

### Loss of Pyroxd1 expression impacts viability of embryos and development of the adult mouse

**Fig. 2:**
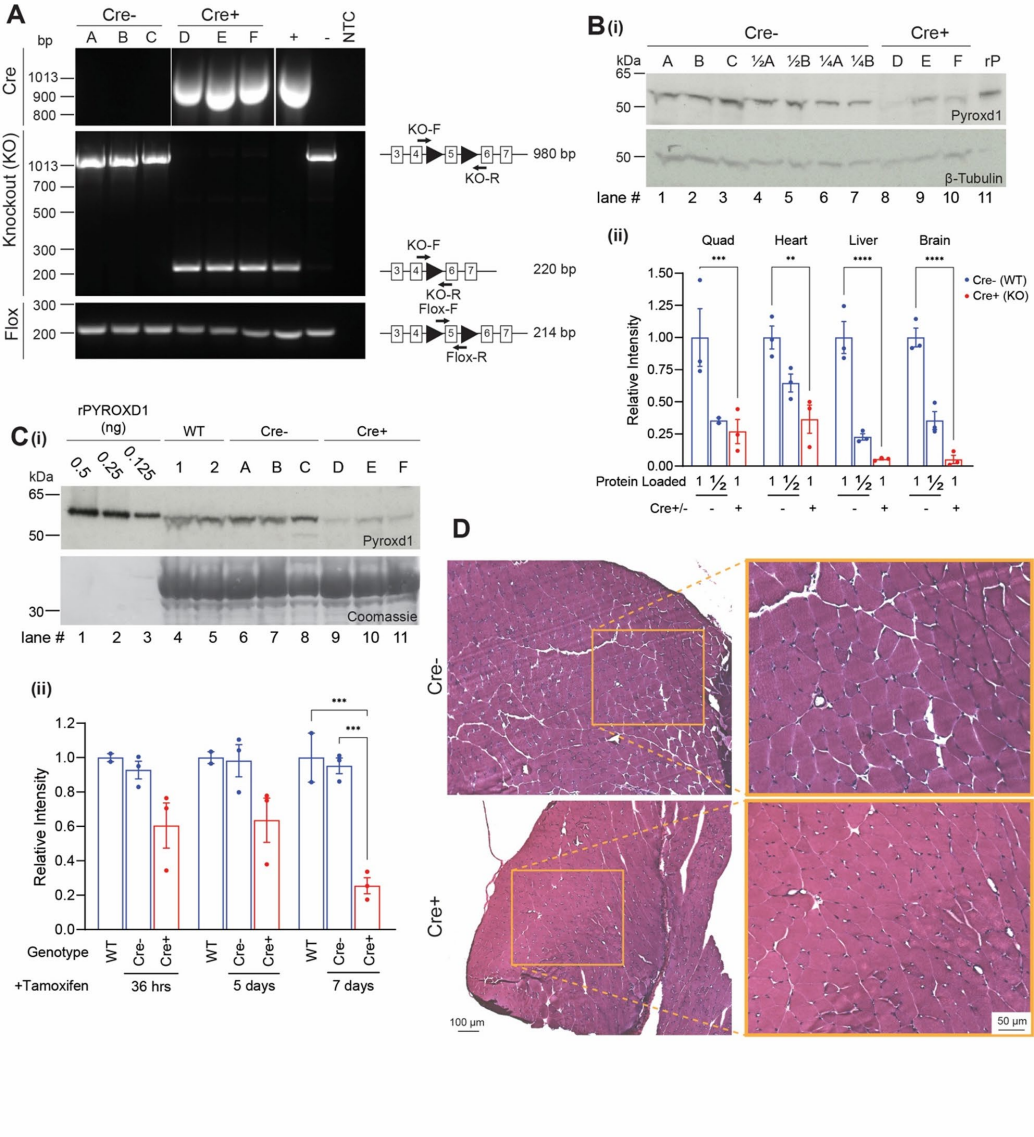
Inducible *Pyroxd1* KO mouse. **A**, **B** Mouse pups were sacrificed at ED18 following tamoxifen administration to pregnant dams at ED14-15 to induce Pyroxd1 KO in Cre + pups in utero. A-F represent six individual mice, three in each condition, representative of n = 39 mice across 5 litters. **A** PCR confirms recombination of LoxP sites around exon 5 in Cre + pups, shown for DNA from skeletal muscle. **B** Western blot demonstrates a reduction in Pyroxd1 protein levels in quadriceps muscle (i) with β-Tubulin included as loading control and relative protein intensities in four tissues quantified (*ii*). Half (½, lanes 4–5) and quarter (¼, lanes 6–7) loading of samples A and B demonstrate the antibody is in the linear range and reductions can be detected. rP (lane 11): purified recombinant human PYROXD1 protein. **C**, **D** 4-week-old Cre- and Cre + mice were injected (n = 3/condition/timepoint) with Tamoxifen on subsequent days, and sacrificed 36 h, 5 days, and 7 days after the second injection. **C** Western blot shows reduced Pyroxd1 protein levels normalised to total muscle protein (Coomassie stain for actin) in quadriceps muscle, shown for 7-days post injection (*i*) with relative intensities quantified (*ii*). Lanes 1–3 decreasing amounts of rPYROXD1: purified recombinant human PYROXD1. **D** H&E staining of quadriceps muscle from 7-days post injection shows no evidence of muscle pathology induced by Pyroxd1 KO, representative images from n = 3 mice/genotype with inset showing magnification for additional detail. **p < 0.01, ***p < 0.001, ****p < 0.0001. See Supplementary Fig. 2 for analysis of additional tissues and timepoints for data presented here

To investigate the effects of conditional loss of *Pyroxd1* activity, we generated a tamoxifen inducible *Pyroxd1* KO mouse model. First, the LacZ reporter cassette in the *Pyroxd1*^Tm1a/WT^ mice was removed by Flippase recombinase to generate mice with floxed *Pyroxd1* allele (PFLP^fl/+^) (Supplementary Fig. 1B*i*) Next, PFLP^fl/+^ mice were crossed with UBC-CreERT2 + mice to generate PFLP^fl/fl^Cre + mice. After intercrossing PFLP^fl/fl^Cre + and PFLP^fl/fl^Cre- mice (Supplementary Fig. 1C), tamoxifen was administered to pregnant dams at ED15, to induce LoxP recombination of *Pyroxd1*to create KO fetuses. Since tamoxifen is known to induce early labour [17], pups were harvested at ED18. PCR genotyping confirmed Mendelian ratios of Cre + /Cre- genotypes and *Pyroxd1* deletion only in Cre + pups (Fig. 2A and Supplementary Fig. 2A). Protein profiling showed significant depletion of PYROXD1 in liver, with a moderate reduction in skeletal muscle, heart, and brain (Fig. 2B and Supplementary Fig. 2B). Contrary to previous reports [5], no concomitant increase in glutathione reductase levels were detected with loss of *Pyroxd1*.

Although late embryonic harvest yielded Pyroxd1 KO tissue, the approach was technically challenging and provided small specimens, insufficient for numerous downstream analyses at scale. So next, we administered two tamoxifen injections on subsequent days to PFLP^fl/fl^ Cre + and PFLP^fl/fl^ Cre- mice at 3–4 weeks of age. Mice were sacrificed at 36 h, 5 and 7 days after their second tamoxifen treatment. By 7 days post tamoxifen, rapid body weight loss (> 10% within 24 h) was observed in 3 out of 5 Cre + mice and mice were not aged any further. PCR confirmed complete excision of the *Pyroxd1* allele in skeletal muscle of Cre + mice by 36 h after tamoxifen administration (Supplementary Fig. 2C). On Western blot, Pyroxd1 protein levels in skeletal muscle were reduced to 60% (36 h–5 days) and 20% (7 days) of wild type (WT) levels (Fig. 2C and Supplementary Fig. 2D). Histology of quadriceps, heart and brain of tamoxifen induced *Pyroxd1* KO mice revealed no obvious pathology in skeletal muscles at 36 h, 5 or 7 days post tamoxifen treatment (Fig. 2D). No compensatory upregulation of glutathione reductase, thioredoxin, thioredoxin reductase or peroxiredoxin was observed in skeletal muscle at any time point (data not shown). Histology of liver from both tamoxifen injected Cre- WT and Cre + KO mice showed minor tamoxifen related toxicity (data not shown).

### Acute Pyroxd1 KO in cultured cells causes a proliferation defect and impaired XBP1 splicing

**Fig. 3:**
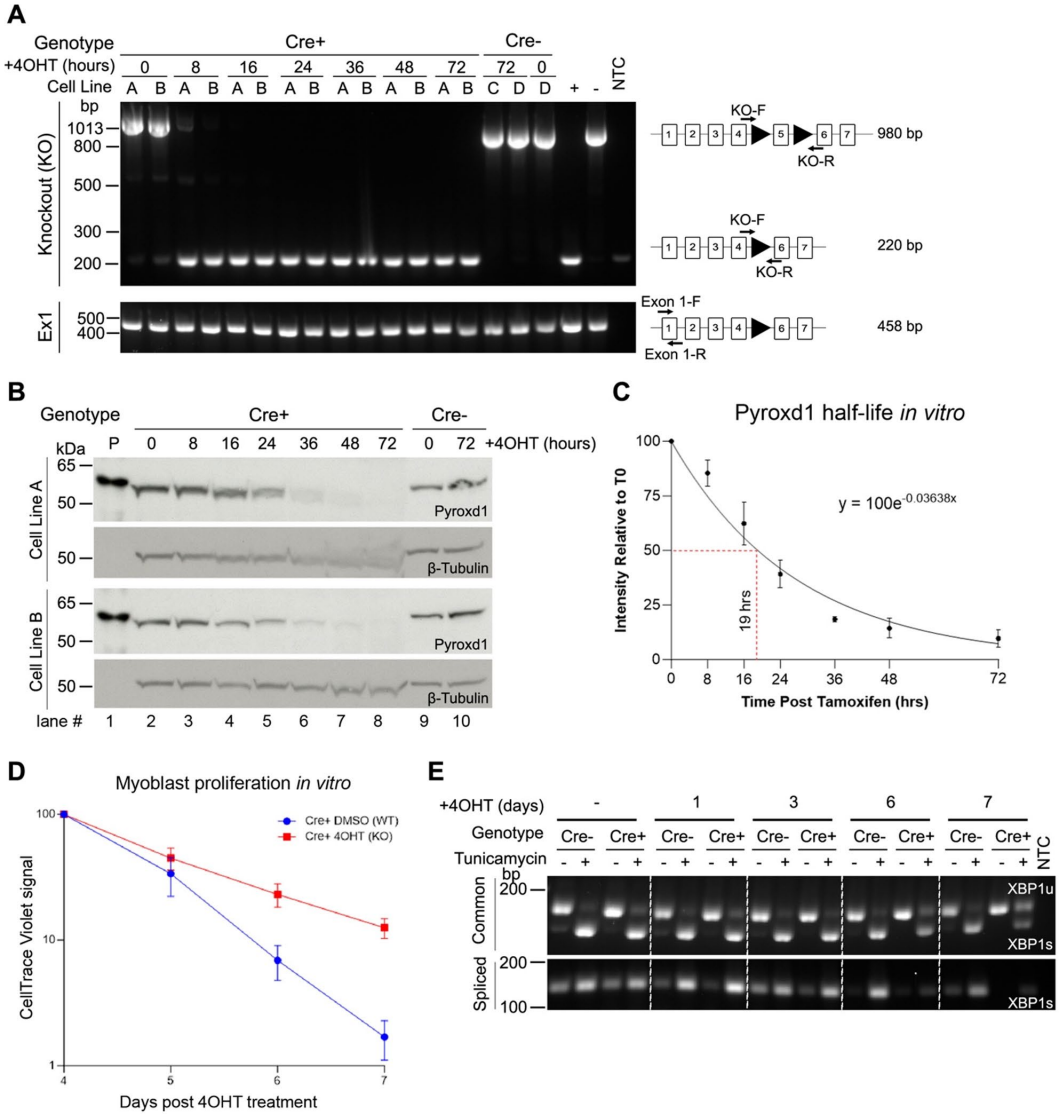
Acute *Pyroxd1* KO in PFLP^+/+^ Cre + myoblasts. **A** PCR screening of Cre + and Cre- myoblasts treated with 4OH-tamoxifen (4OHT) for 0–72 h showing efficient recombination of the floxed *Pyroxd1* exon 5 allele only in Cre + myoblasts. A, B, C and D refer to independently derived cell lines **B** Western blot shows loss of Pyroxd1 protein in Cre + myoblasts by 36 h of 4OHT treatment with β-Tubulin as a loading control. Lane 1, P = purified recombinant PYROXD1 protein loaded as a control to show the correct identity of the observed bands. **C** Normalised Pyroxd1 protein half-life plotted from the quantification of western blot in (**B**) and additional replicates. **D** Cell trace violet assay in PFLP^+/+^ Cre + and Cre- proliferating myoblasts treated with 4OHT with cell trace violet integrated on day 4 after 4OHT treatment. Cell trace violet signal was normalised and quantified up to 7 days post 4OHT treatment to detect changes in the rate of cell division. See methods for details. **E** PFLP^+/+^ Cre + and Cre- myoblasts were treated with 4OHT for 1, 3, 6 or 7 days then treated on the last day with Tunicamycin to activate the unfolded protein response. RT-PCR of X-Box Protein 1 (*XBP1*) non-canonical splicing is used as a proxy for detection of UPR activation (top: common primers detecting both forms, bottom: spliced form specific primers). XBP1s: spliced and ligated XBP1, XBP1u: unspliced XBP1

We leveraged our *Pyroxd1* mouse models to derive inducible *Pyroxd1* knockout primary myoblast cell lines from 3 to 5-day-old Cre- and Cre + PFLP^+/+^ pups, hereafter referred to as Cre + or Cre- myoblasts. In Cre + myoblasts, cell-permeable 4-hydroxytamoxifen (4OHT) treatment triggered rapid *Pyroxd1* allele recombination, with no evidence of a non-recombined allele after 16 h (Fig. 3A). Cre- lines served as 4OHT-treated non-recombined controls (Fig. 3A). Western blot confirmed progressive reduction of Pyroxd1 protein expression in Cre + KO myoblasts, with residual levels after 36 h of 4OHT treatment and no Pyroxd1 protein detected at 48 h (Fig. 3B). Analysis of four, independently derived Cre + myoblast cell lines after 4OHT treatment estimates Pyroxd1 half-life to be approximately 19 h (Fig. 3C).

Cre- and Cre + myoblast cell lines without 4OHT treatment grew similarly and spontaneously differentiated into myotubes as expected when allowed to reach confluence. However, when maintained as proliferating myoblasts and treated with 4OHT, Cre + *Pyroxd1* KO myoblasts demonstrated visibly reduced growth after 6 days with an inability to replate following trypsinisation after 7–8 days. Flow cytometry analysis using CellTrace violet fluorescent cell staining confirmed a proliferation defect from day 5 of 4OHT induced KO in Cre + myoblasts (Fig. 3D).

As PYROXD1 regulates the tRNA ligase complex [1, 14], which mediates non-canonical *XBP1* splicing [18] to activate the unfolded protein response (UPR), we assessed tunicamycin-triggered *XBP1* splicing following Pyroxd1 KO. No differences in *XBP1* splicing was observed for up to 5 days post 4OHT treatment, but at day 6 and more clearly day 7 of 4OHT treatment, reduced *XBP1* splicing was detected only in tunicamycin-treated KO (Cre +) myoblasts (Figured 3E).

### Creation of the first mouse model of PYROXD1 myopathy, homozygous Pyroxd1_N155S_

To study the pathobiology of human *PYROXD1* myopathy, we created a mouse model of the recurrent [7] human *PYROXD1* variant p.N155S. An additional model, p.N155G was created incidentally during CRISPR base editing – being potentially useful for variant interpretation, both homozygous models were studied. *Pyroxd1*_N155S_ and *Pyroxd1*_N155G_ mice are viable and can be bred at homozygosity.

Given the relatively mild phenotype of humans homozygous for the p.N155S variant and that many individuals are compound heterozygous for p.N155S and a loss of function variant [3, 7], we also aimed to create a compound heterozygous *Pyroxd1*_N155S/KO_ mouse (see methods) to ensure we could observe a phenotype. After multiple rounds of breeding, we determined *Pyroxd1*_N155S/KO_ mice were also embryonic lethal, with no viable *Pyroxd1*_N155S/KO_ pups born or embryos remaining across four litters at mid-gestation (ED9-ED15) and signs of embryo resorption. Breeding of *Pyroxd1*_N155S/KO_ mice was thus discontinued to focus on homozygous *Pyroxd1*_N155S_ and *Pyroxd1*_N155G_ lines.

**Fig. 4:**
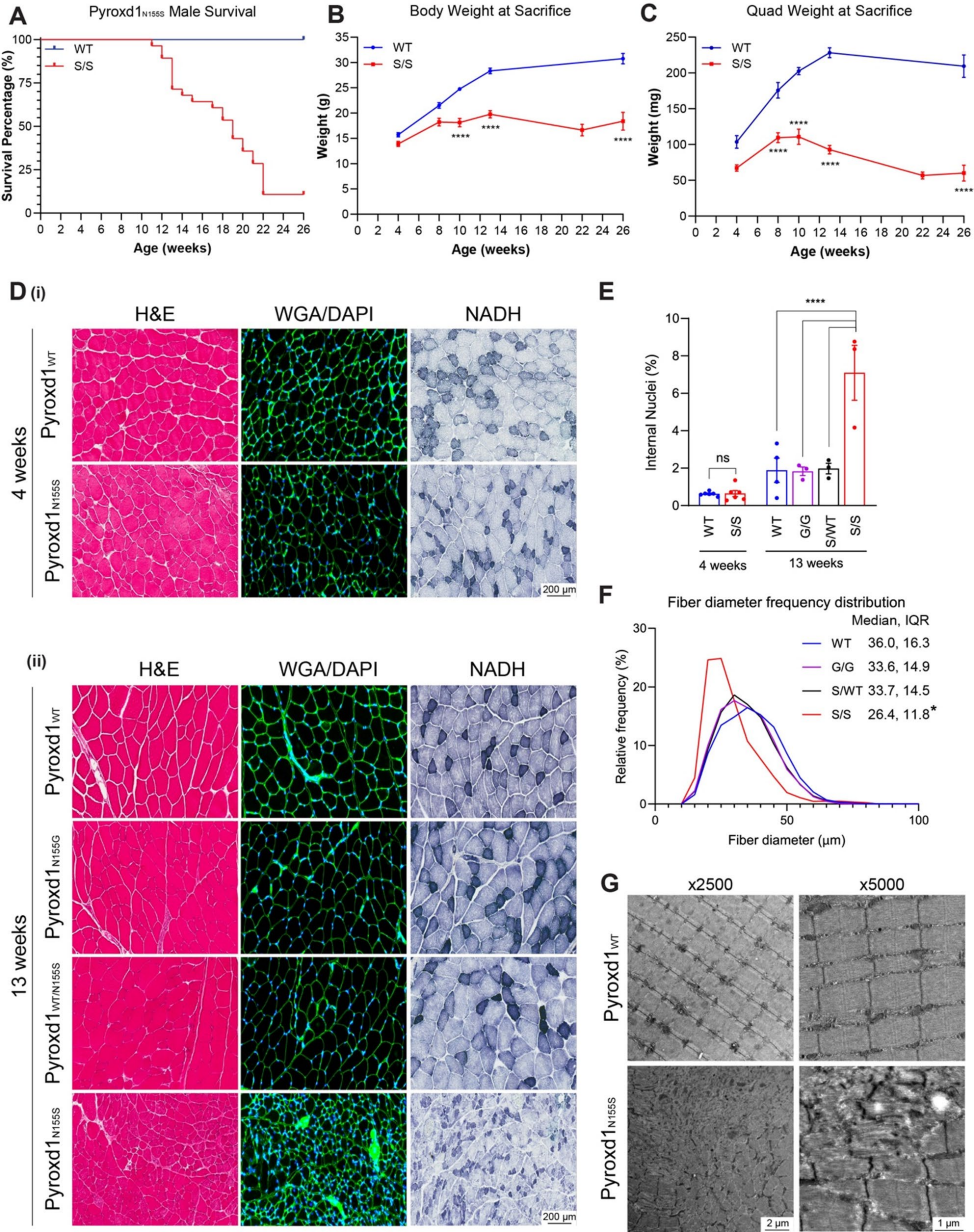
*Pyroxd1*_N155S_ mice have a measurable skeletal muscle phenotype. Comparisons of gross size and microstructure in muscles from each genotype as mice age **A** Survival curve of *Pyroxd1*_WT_ (n = 28) versus *Pyroxd1*_N155S_ (n = 28) mice up to 26 weeks of age. Total body (**B**) and quadriceps muscle weights **C** were measured over the same period from tissue harvested mice (n = 5–7 mice/genotype/age group). **D** H&E, WGA/DAPI, and NADH staining of quadriceps muscles at 4 (*i*) and 13 (*ii*) weeks of age. Each panel is a representative sample from n = 3 mice/genotype/age group. **E** Quantification of percentage of internalised nuclei in WGA/DAPI-stained quadriceps muscles (n = 3–6). **F** Fibre diameter distribution of WGA/DAPI stained 13-week-old mouse quadriceps muscles (n = 3–6). **G** TEM images of longitudinally oriented quadriceps muscles showing normal sarcomeric structure in *Pyroxd1*_WT_ mice and sarcomeric disruption in 16–17-week-old *Pyroxd1*_N155S_ mice. *p < 0.05, ****p < 0.0001). WT: *Pyroxd1*_WT_, S/S: *Pyroxd1*_N155S_, G/G: *Pyroxd1*_N155G_, S/WT: *Pyroxd1*_WT/N155S_

*Pyroxd1*_N155S_ mice litters were consistently small, often only 2–4 pups. Further, when aging mice for phenotypic characterisation, we observed sudden death of *Pyroxd1*_N155S_ males from 10 weeks of age, with an increasing incidence from 14 weeks (Fig. 4A). Affected males were under-weight but otherwise healthy before becoming acutely unwell, with grimace, immobility and shaking, sometimes coupled with rapid weight loss, before humane intervention. No sudden unexplained death was observed in female *Pyroxd1*_N155S_ mice. Autopsies (Cerberus Sciences, Thebarton, SA, Australia) were conducted for male mice near 10 weeks of age that were stable, acutely sick, or recently deceased from a sudden death (Supplementary Fig. 3A). This revealed skeletal muscle pathology and testicular degeneration of stable *Pyroxd1*_N155S_ males (not shown), with urethral blockages and multifocal haemorrhage in the bladder (Supplementary Fig. 3B), along with dilated renal tubules and ureters (Supplementary Fig. 3C) in acutely sick and deceased *Pyroxd1*_N155S_ males. This mirrors the pathology described in the *Acta1* H40Y mouse model of nemaline myopathy linked to early death of male mice [19, 20]. We thus propose a similar, male murine-specific bladder outlet obstruction due to muscle weakness (smooth and/or skeletal) that causes sudden death in male *Pyroxd1*_N155S_ mice but this has not been functionally explored in the current study. For ethical reasons, after initial characterisation male *Pyroxd1*_N155S_ mice were not aged beyond 16 weeks, with female mice used for older time points.

Although the use of precise base editing argues against off-target effects of CRISPR editing contributing to the observed phenotype in *Pyroxd1*_N155S_ mice, confirmatory whole genome sequencing (WGS) was performed. Due to multi-generational breeding selecting for the N155S variant, the only plausible off-target possibility would be a homozygous variant *in cis* with the variant *Pyroxd1* allele. Comparative WGS from *Pyroxd1*_WT,_ *Pyroxd1*_N155S_ and *Pyroxd1*_N155G_ mice did not identify any additional plausible homozygous variants among 61,108 variants in any genes associated with severe murine pathologies, other than the targeted substitution of *Pyroxd1* Asn155 (Supplementary Fig. 3D).

### Pyroxd1_N155S_ mouse model phenocopies human PYROXD1 disease skeletal muscle presentation

Longitudinal analysis of *Pyroxd1*_N155S_ mice over 6 months for males and 12 months for females showed *Pyroxd1*_N155S_ mice were considerably smaller than WT mice (Supplementary Fig. 4A), with distinct postural changes when hind limb rearing and splayed back legs during handling (Supplementary Fig. 4B). From 10 weeks of age, at sacrifice, *Pyroxd1*_N155S_ mice weighed 25—40% less than their WT littermates (Fig. 4B, Supplementary Fig. 4C) due primarily to a progressive 38 -71% reduction in skeletal muscle weight from 8 weeks of age (shown for quadriceps and gastrocnemius, Fig. 4C, Supplementary Fig. 4D-E). Importantly, tibia lengths (Supplementary Fig. 4F) and organ weights (Supplementary Fig. 4G) were not significantly different between *Pyroxd1*_N155S_, *Pyroxd1*_N155G_ and *Pyroxd1*_WT_ mice, excluding a global growth defect.

Skeletal muscle histology was assessed with H&E staining for overall structure, wheat germ agglutinin and DAPI (WGA/DAPI) discerning fibre size and presence of internalised nuclei, and NADH staining showing mitochondrial activity distribution (Fig. 4D). At 4 weeks of age, *Pyroxd1*_WT_ and *Pyroxd1*_N155S_ mice showed relatively normal histology (Fig. 4Di, E). However, by 13 weeks of age, *Pyroxd1*_N155S_ mice showed a profound muscle pathology compared to *Pyroxd1*_WT_, *Pyroxd1*_WT/N155S_ or *Pyroxd1*_N155G_ mice (Fig. 4Dii). Features include increased internalised nuclei (Fig. 4Dii, E), uneven NADH staining (Fig. 4Dii) and reduced fibre diameter (Fig. 4Dii, F) compared to all control genotypes. Electron microscopy revealed significant sarcomeric disruption in *Pyroxd1*_N155S_ mouse skeletal muscle, including areas with profoundly disrupted myofibrillar architecture and presence of nemaline rods (Fig. 4G). These histopathological features phenocopy those seen in humans with PYROXD1 myopathy [3, 5].

### Pyroxd1_N155S_ mouse muscle fibre typing reveals a shift towards smaller type 2B fibres

**Fig. 5:**
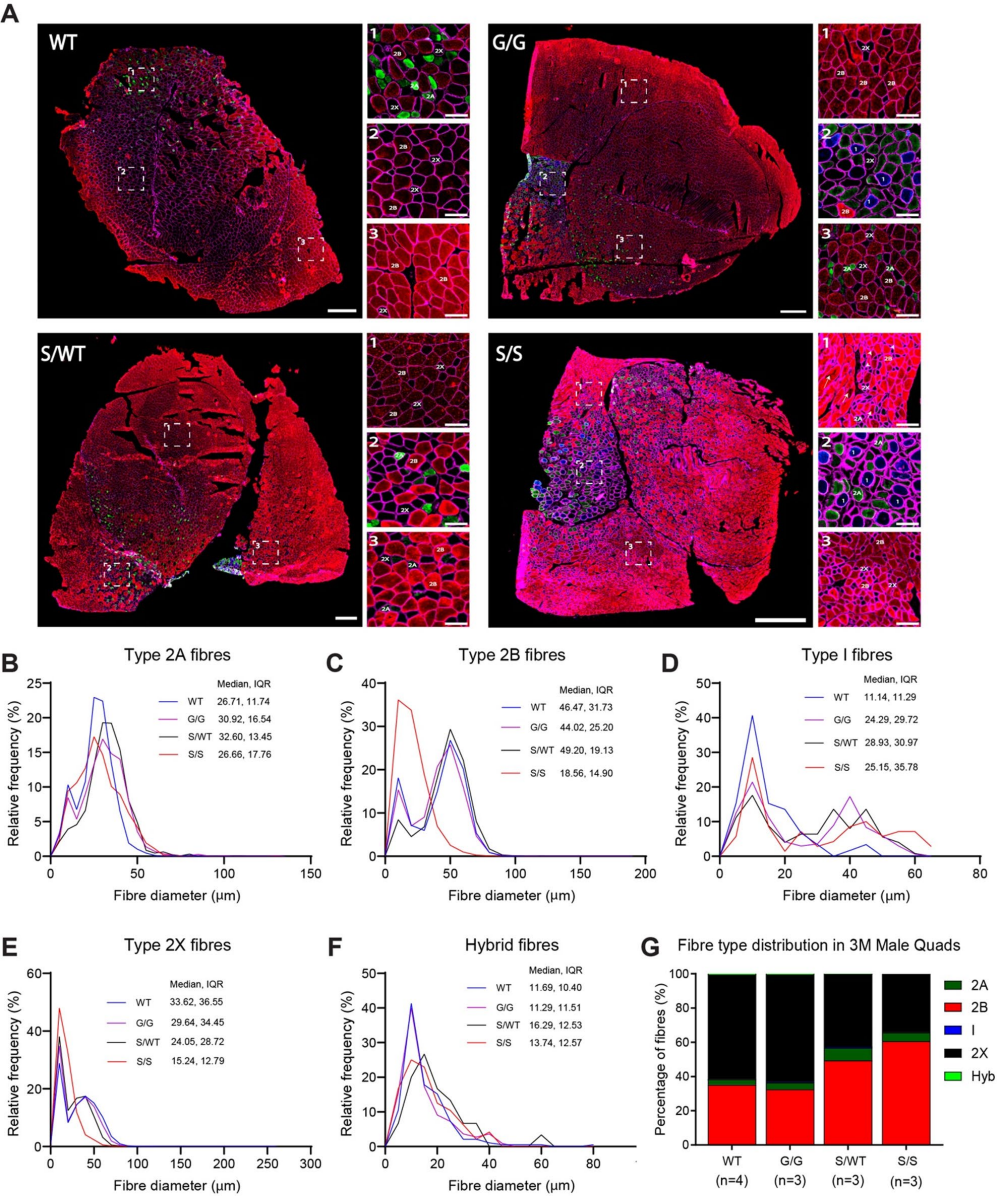
*Pyroxd1*_N155S_ mice display a fibre type shift with smaller intensely stained type 2B fibres. **A** Representative images of quadriceps muscle sections from 3-month-old wildtype (WT, n = 4), *Pyroxd1*_N155G_ (G/G, n = 3), *Pyroxd1*_N155S/WT_ (S/WT, n = 3) and *Pyroxd1*_N155S_ (S/S, n = 3) mice. Sections co-stained for dystrophin (to mark the sarcolemma, magenta), myosin heavy chain type 1 (blue), type 2A (green) and type 2B (red) fibres. Type 2X fibres (black) are unstained, hybrid fibres stain for markers of two or more fibre types. S/S mice, show a predominance of smaller dark red stained type 2B fibres, with some fibres showing a patchy staining pattern possibly indicative of internal cores (arrows) and areas of thickened sarcolemma (arrow heads). Scale bars are 500 µm in whole muscle images and 100 µm in magnified images. **B–F** Frequency distribution histogram of muscle fibre diameter, determined by a custom-made Image J macro, across entire muscle sections are shown for each fibre type and compared across the four genotypes. The median fibre diameter (MFD) and interquartile range (IQR) are shown at the top right of each graph. One-way ANOVA followed by Kruskal–Wallis multiple comparison test revealed no significant difference in MFD between S/S and WT mice in all fibre types, however, S/S mice had notably have smaller type 2B fibres as shown in C. **G** Quantification of percentage of fibre types across the muscle section reveals a shift from type 2X to type 2B fibres in S/S mice. *Pyroxd1*_WT_ and *Pyroxd1*_N155G_ mice: type 2A fibres 3.1 ± 0.8%, type 2B 35.1 ± 4.3%, type 2X 60.1 ± 4.9%, type I and hybrid fibres accounting for less than 1% of the total number of fibres. There was a slight increase in the proportion of type 2A fibres in both *Pyroxd1*_N155S/WT_ mice (7.5 ± 3.2%) and *Pyroxd1*_N155S_ mice (5.1 ± 1.5%). The percentage of type 2B fibres was significantly increased in *Pyroxd1*_N155S_ mice (60.6 ± 3.8% vs 35.1 ± 4.3% in WT, **p = 0.0083). A trend towards an increased proportion of type 2B fibres in *Pyroxd1*_N155S/WT_ mice was also noted (49.4 ± 7.4%, p = 0.1375). Compared to *Pyroxd1*_WT_ mice (60.1 ± 4.9%), the percentage of type 2X fibres was reduced to 42.4 ± 5.7% (p = 0.0559) in *Pyroxd1*_N155S/WT_ mice and ~ 33.8 ± 2.2% (**p = 0.0067) in *Pyroxd1*_N155S_ mice, suggesting a fibre type shift from type 2X towards type 2B in *Pyroxd1*_N155S/WT_ and *Pyroxd1*_N155S_ mice. M = age in months, Hyb = Hybrid fibres

Mouse skeletal muscle is composed of four myosin heavy chain (MHC) fibre types with distinct contractile and metabolic properties, namely, type 1 (slow oxidative), type 2A (fast oxidative), type 2B and type 2X (fast glycolytic) [21]. At 12 weeks of age, *Pyroxd1*_WT_*, Pyroxd1*_N155G_ and *Pyroxd1*_N155S/WT_ quadriceps muscle showed similar fibre type distributions, dominated by red stained type 2B fibres and unstained type X fibres, with very few type I and 2A fibres (Fig. 5A). *Pyroxd1*_N155S_ muscle uniquely exhibited numerous internal cores within 2B fibres devoid of antibody staining (white arrowhead, Fig. 5A, S/S inset 1), and thickened sarcolemma in areas of tightly packed fibres (white arrow, Fig. 5A, S/S inset 1).

The size and relative abundance of type 2A, type 1, and hybrid fibres was broadly similar across the four genotypes (Fig. 5B, D and F), with only subtle changes observed in *Pyroxd1*_N155S_ mice. However, *Pyroxd1*_N155S_ quadriceps muscles showed clear abnormalities in relative abundance and sizes of 2B and 2X fibres. While *Pyroxd1*_WT_, *Pyroxd1*_N155G,_ and *Pyroxd1*_N155S/WT_ muscle showed a bimodal size distribution of 2B and 2X fibres (Fig. 5C, E), *Pyroxd1*_N155S_ muscle showed a left-shifted, unimodal distribution toward smaller diameter fibres, particularly for 2B fibres (Fig. 5C, E). *Pyroxd1*_N155S_ muscle had a higher proportion of 2B fibres (60.6% versus 35.1% in WT, p = 0.0083) and reduced relative numbers of 2X fibres (~ 33.8% versus 60.1% in WT, p = 0.0067). Therefore, impaired Pyroxd1_N155S_ function activates an apparent shift from type 2X to 2B fibres (Fig. 5G). Despite reduced overall muscle mass and fibre diameter (Fig. 4C, F), the total number of fibres in *Pyroxd1*_N155S_ quadriceps was comparable to other genotypes, resulting in an increased fibre density (Supplementary Fig. 5). Interestingly, heterozygous *Pyroxd1*_N155S/WT_ mice showed a similar trend toward an increased proportion of type 2B fibres and reduced 2X fibres, whereas no overt changes in fibre size or type were observed in *Pyroxd1*_N155G_ compared to WT muscles (Fig. 5G).

### Pyroxd1_N155S_ mice display a predominantly fast muscle contractile dysfunction

**Fig. 6:**
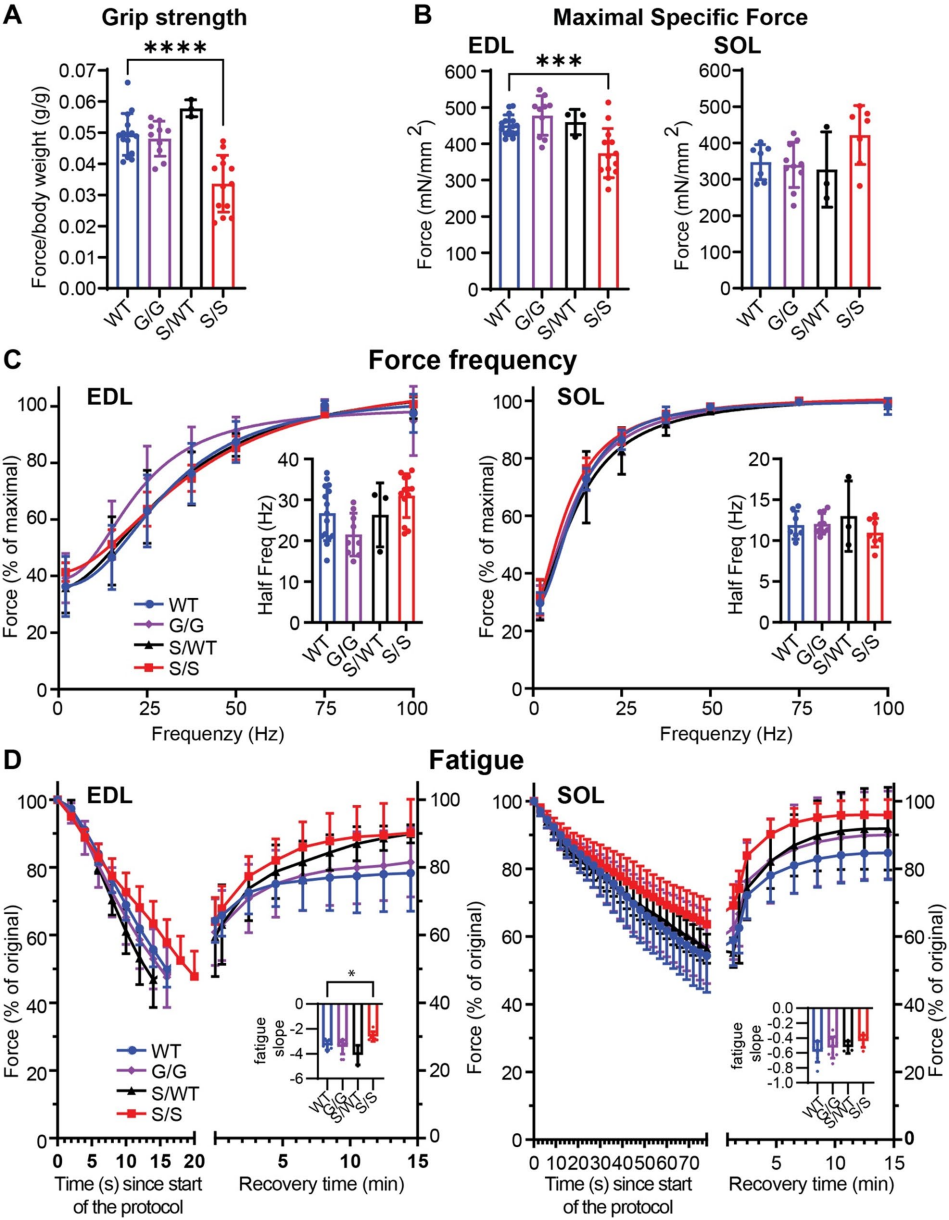
*Pyroxd1*_N155S_ mice display fast twitch skeletal muscle weakness. **A** Forelimb grip strength performed on 14-week-old WT (*Pyroxd1*_WT_), S/S (*Pyroxd1*_N155S_), G/G (*Pyroxd1*_N155G_) and S/WT (*Pyroxd1*_WT/N155S_) mice. Grip strength normalised to mouse body weight was 31.92% reduced in S/S mice compared to WT (WT: 0.049 ± 0.007, S/S 0.058 ± 0.003; ****p < 0.0001) while G/G and S/WT mice showed grip strength similar to WT mice. **B** Ex vivo maximal specific tetanic force of EDL (left) and SOL muscles (right). The contractile performance of EDL and SOL were normalised to muscle cross sectional area (CSA) to obtain specific forces and account for observed reduction in muscle size. Significant force deficit was only found between S/S EDL and WT EDL (374.3 ± 67.8 mN/mm^2^ vs. 450.8 ± 28.5 mN/mm^2^, p = 0.0009***). No significantly different forces were measured in SOL. A trend towards increased specific force in S/S SOL compared to other genotypes was observed (WT: 347.3 ± 48.4 mN/mm^2^, G/G: 339.8 ± 62.4 mN/mm^2^, S/WT: 326.9 ± 103.7 mN/mm^2^, S/S: 421.9 ± 81.2 mN/mm^2^). **C** Force/Frequency curves. **D** Fatigue and recovery curves of EDL and SOL with fatigue slope presented in the inset bar graph. The number of contractions to achieve 50% force loss: 8 in EDL of WT, G/G, S/WT and 10 in EDL of S/S mice; 26 in SOL of all genotypes. After a 15 min recovery period, EDL muscles achieved 78.3% ± 11.2% (WT; n = 7), 81.5% ± 10.3% (G/G; n = 10), 89.9% ± 2.7% (S/WT; n = 3) and 90.2% ± 9.9% (S/S; n = 7) of their initial force, and SOL muscles recovered 84.7% ± 7.9% (WT; n = 7), 90.1% ± 13% (G/G; n = 10), 91.9% ± 12.2% (S/WT; n = 3) and 95.9% ± 4.5% (S/S; n = 7). See Supplemental Fig. 6 for force comparisons of all genotypes in EDL and SOL at various stages of the fatigue protocol. Error bars show standard deviations. One-way ANOVA comparing WT to all other genotypes was performed and statistically significant results (*p < 0.05) are indicated

To evaluate the contractile function of *Pyroxd1*_N155S_ muscles, we performed in vivo forelimb grip strength testing in 14-week-old mice, followed by ex vivo whole hindlimb muscle contractility analyses of the predominantly fast glycolytic extensor digitorum longus (EDL) and slow twitch soleus (SOL) muscles.. *Pyroxd1*_N155S_ mice exhibited significantly reduced fore limb grip strength compared to *Pyroxd1*_WT_ controls, even when normalised to muscle mass (32% reduction, ****p < 0.0001, Fig. 6A). No significant force deficit relative to muscle mass was observed in other genotypes.

Consistent with our characterisation of *Pyroxd1*_N155S_ quadriceps and gastrocnemius muscles (Fig. 4 & Supplementary Fig. 4), *Pyroxd1*_N155S_ fast glycolytic tibialis anterior (TA) and EDL muscles were disproportionately small (muscle weight/body weight; ****p < 0.0001; TA -34.5%, EDL –21.3%, Supplementary Fig. 6Aii-iii), while the slow twitch SOL muscles of *Pyroxd1*_N155S_ were proportionally similar in mass compared to overall body weight (Supplementary Fig. 6Ai) and WT controls. This suggests the slow SOL muscle is comparatively spared in *Pyroxd1*_N155S_ mice. Muscle size was generally unaffected in *Pyroxd1*_N155G_ and *Pyroxd1*_N155S/WT_ mice compare to *Pyroxd1*_WT_ mice, though TA and EDL muscles were slightly smaller in (Supplementary Fig. 6Aii-iii).

Ex vivo specific force measurements during a maximal tetanic contraction were reduced by 17% in *Pyroxd1*_N155S_ EDL muscle compared to *Pyroxd1*_WT_ EDL (***p = 0.0009; Fig. 6B), whereas SOL specific force was comparable between genotypes (Fig. 6B). The force-frequency relationship was unchanged in *Pyroxd1*_N155S_ muscles and other genotypes, indicating preserved excitation/contraction coupling (Fig. 6C).

To assess muscle fatigue, repeated force stimulations were applied to SOL and EDL muscles until force declined by ~ 50%, followed by a 15 min recovery period and restimulation to assess muscle recovery post fatigue (Fig. 6D and Supplementary Figs. 7 Aii and Bi). Unexpectedly, the fatigue force loss slope was significantly steeper in EDL of *Pyroxd1*_WT_ compared to *Pyroxd1*_N155S_ mice (*p = 0.0371; Fig. 6D inset) indicating *Pyroxd1*_N155S_ EDL were fatigue resistant. After a 15-min recovery period similar force recovery kinetics were observed across genotypes (Supplementary Fig. 7Aiii and Bii).

Passive stiffness of both EDL and SOL was determined using a viscoelastic model separating “in series” and “in parallel” elastic elements. No marked differences were detected between genotypes during a 5% stretch (Supplementary Fig. 8).

Eccentric contraction testing (10 eccentric contractions with 10% stretch of 1 L_O_/s during a maximal tetanic contraction) revealed steeper initial force loss in *Pyroxd1*_N155S_ EDL (*p = 0.0353) compared to *Pyroxd1*_WT_ and other genotypes with no significant difference in end forces in SOL consistent with the known susceptibility of fast muscles to eccentric damage (Supplementary Fig. 9A–B). To further interrogate the mechanisms underlying force decline, active stiffness measurements were used to calculate the proportion of attached cross bridges during eccentric contractions (Supplementary Fig. 9C and D). This analysis demonstrated a significant increase in the fraction of active cross bridges in EDL from *Pyroxd1*_N155S_ mice (62.6 ± 5.8%) compared to *Pyroxd1*_WT_ mice (57.0 ± 4.5%) and other groups (**p = 0.0087), whereas no differences was observed in SOL.

Collective muscle physiology data consistently indicate fast fibres are more vulnerable to impaired *Pyroxd1*_N155S_ function than slow fibres.

### Pyroxd1_N155S_ mice have a measurable bone phenotype but no other connective tissue anomalies

**Fig. 7:**
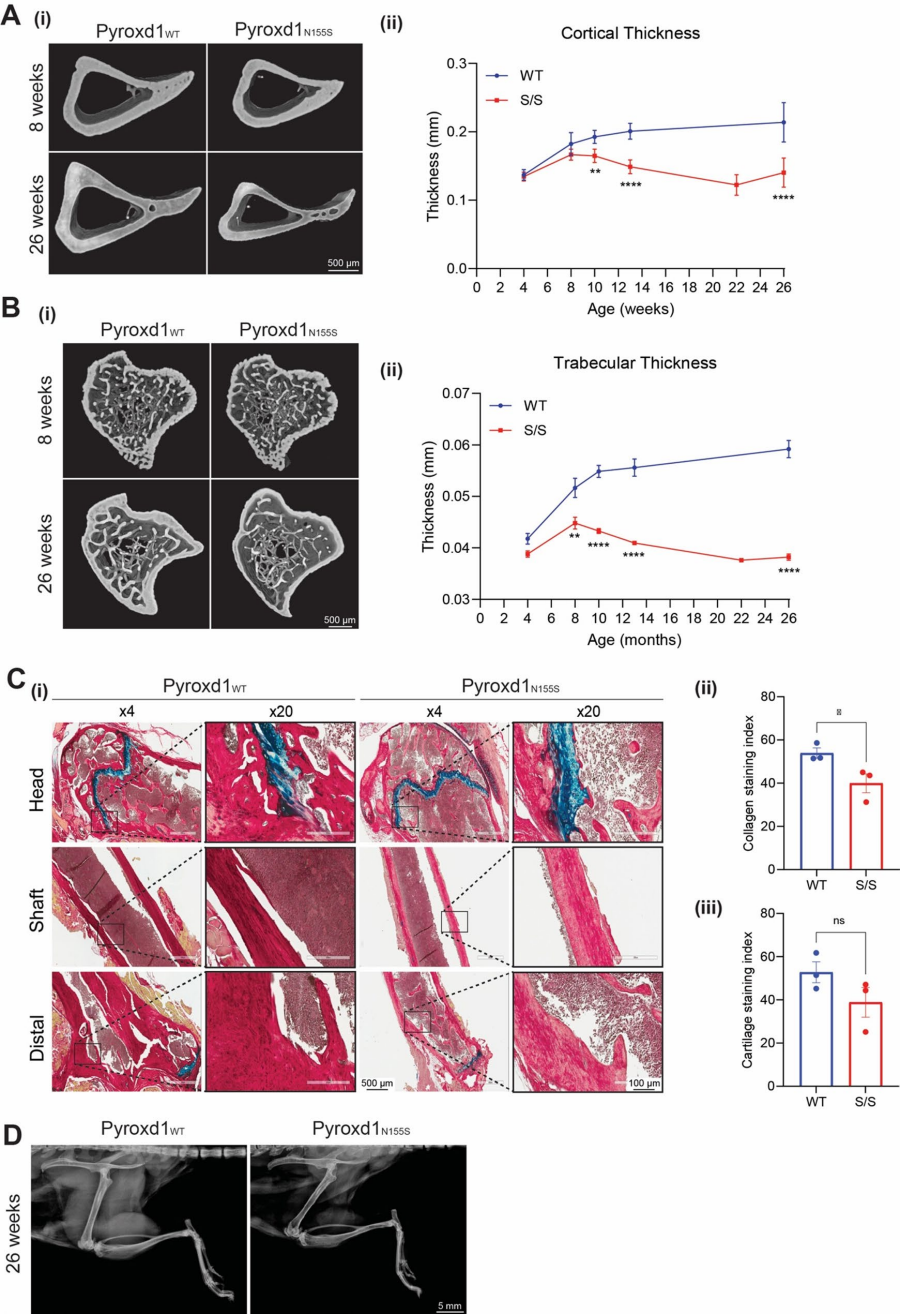
*Pyroxd1*_N155S_ mice display a bone phenotype with reduced bone volume and reduced collagen deposition. Bone analysis of *Pyroxd1*_WT_ and *Pyroxd1*_N155S_ mice. **A** MicroCT shows reduced tibia cortical thickness and **B** reduced trabecular thickness and complexity in *Pyroxd1*_N155S_ mice (n = 6–8 mice/genotype/ time point) **C** (*i)* Alcian blue (blue, cartilage) and Picrosirius Red (red, Collagen) staining of longitudinal bone sections showing reduced collagen staining and non-significant cartilage reduction in *Pyroxd1*_N155S_ mouse bones compared to *Pyroxd1*_WT_, quantified in (*ii)*. n = 3/genotype. **D** Whole body X-ray reveals no spontaneous bone breakages in *Pyroxd1*_WT_ or *Pyroxd1*_N155S_ mice (WT n = 12, NS n = 31, hind limb X-ray shown)

Given the recent expansion of the PYROXD1 disease phenotype to include connective tissue features [7], we investigated potential bone and connective tissue changes in *Pyroxd1*_N155S_ mice. Tibia microCT imaging revealed comparable cortical thickness between all genotypes up to 8 weeks of age, followed by significant cortical thinning in *Pyroxd1*_N155S_ mice from 10 weeks of age (Fig. 7A). Trabecular thinning was observed in *Pyroxd1*_N155S_ mice from 8 weeks of age (Fig. 7B), preceding cortical thinning. Extended microCT analysis confirmed significant reductions in cortical volume, polar mean moment of inertia (a proxy for bone strength), trabecular bone mineral density, and bone volume relative to tissue volume in *Pyroxd1*_N155S_ mice from 10 weeks of age (Supplementary Fig. 10A). Alcian blue and Picrosirius Red staining (Fig. 7Ci) revealed significantly reduced total collagen deposition and collagen staining intensity along the length of the tibia (Fig. 7Cii), with a non-significant reduction in cartilage thickness and staining intensity (Fig. 7Ciii). Despite these indicators of compromised bone quality in *Pyroxd1*_N155S_ mice, no spontaneous fractures were observed, as confirmed by whole body X-ray at sacrifice (Fig. 7D, representative image from age 26 weeks).

To further assess connective tissue integrity, we performed electron microscopy of collagen fibrils in eye, skin and aorta to assess for abnormal collagen fibril organisation often seen in primary connective tissue disorders [22]. Due to variability in the available eye sections, detailed analyses were not feasible. However, quantitative assessment of > 1000 individual collagen fibrils in skin and aorta from 13-week-old male *Pyroxd1*_N155S_ and WT mice revealed no significant alteration of skin and aorta collagen fibrils size or shape (Supplementary Figs. 10B and C).

### Opposing proteomic signatures in Pyroxd1 knock out and Pyroxd1_N155S_ models

**Fig. 8:**
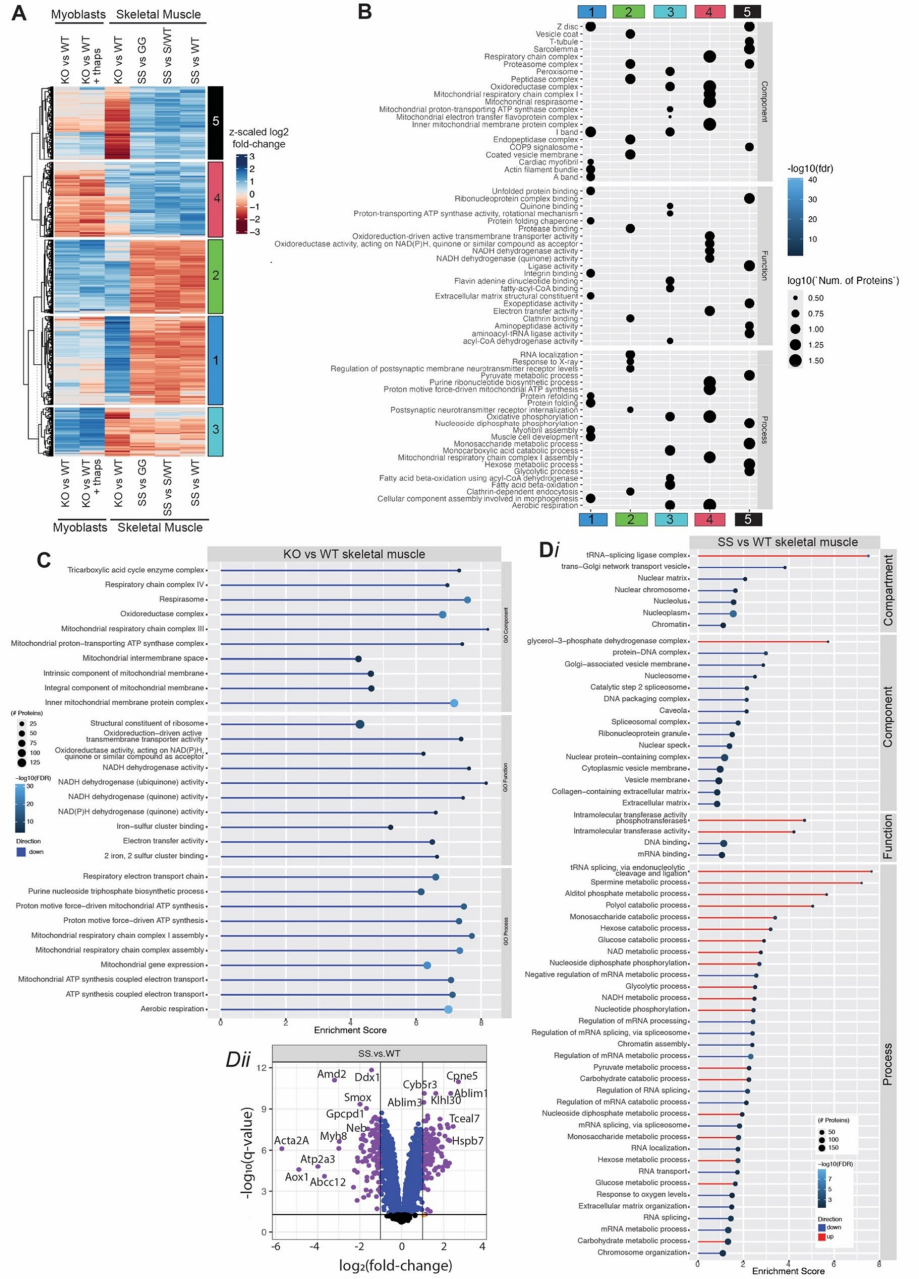
Comparative proteomic analysis of Pyroxd1 inducible KO myoblasts, KO skeletal muscle and *Pyroxd1*_N155S_ muscle. **A** Heat map showing up and down regulated proteins across all proteomic comparisons. **B** GO functional enrichment of protein groups in (**A**). **C** GO functional enrichment pathway analysis of *Pyroxd1* KO skeletal muscle compared to *Pyroxd1* WT skeletal muscle. **D** *(i)* GO functional enrichment pathway analysis of *Pyroxd1*_N155S_ mouse skeletal muscle compared to *Pyroxd1*_WT_ mouse skeletal muscle. (*ii)* Volcano plots showing significantly up and down regulated proteins in *Pyroxd1*_N155S_ mouse skeletal muscle compared to *Pyroxd1*_WT_ mouse skeletal muscle. The most significantly down regulated proteins include Acta2 (actin α2, smooth muscle actin), AOX1 (molybdenum binding enzyme), Atp2a3 (SERCA Ca^2+^-ATPases), ABcc12 (ATP-binding cassette sub-family A member 12, membrane transporter), Amd2 (S-adenosylmethionine decarboxylase proenzyme 2, essential for biosynthesis of the polyamines spermidine and spermine), Myh8 (myosin heavy chain 8), Bpnt2 (3'(2'), 5'-Bisphosphate Nucleotidase 2, a member of the inositol monophosphatase family) and Phkb (phosphorylase kinase regulatory subunit beta, regulates activity of Phosphorylase b kinase involved in glycogen breakdown). The most significantly upregulated proteins include Cpne5 (Copine-5, calcium-dependent phospholipid-binding protein), Mlf2 (myeloid leukemia factor 2, involved in the regulation of DNA-templated transcription), Ablim1 (actin-binding LIM protein 1), Hspb7 (Heat shock protein beta-7, highly expressed in heart and skeletal muscle), Tceal7 (transcription elongation factor A like 7, negative regulation of DNA-templated transcription) and Xirp2 (Xin Actin Binding Repeat Containing 2). For additional proteomic comparisons and data see Supplementary Figs. 11–13

Comparative proteomics was performed to gain mechanistic insight into the consequences of acute *Pyroxd1* KO (skeletal muscle and cultured myoblasts) or expression of Pyroxd1 with reduced function, *Pyroxd1*_N155S_ (skeletal muscle). Data was normalised using the RUVIII method and principal component analyses (PCA) showed appropriate clustering of replicates (Supplementary Figs. 11A, 12A, 13A). The String DB functional enrichment tool [23] was used to categorise gene ontology (GO) clusters into five groups (Fig. 8A, B). The heat map in Fig. 8A shows opposing proteomic signatures between *Pyroxd1* deficiency and expression of the *Pyroxd1*_N155S_ variant for most GO clusters, with cluster compositions shown in Fig. 8B.

In cultured myoblasts, acute *Pyroxd1* KO led to widespread and profound dysregulation of multiple oxidative and reductive enzymatic pathways (Fig. 8A, B). Group 3, comprising proteins involved in aerobic respiration, fatty acid oxidation, oxidative phosphorylation and catabolism of monocarboxylic acids (lactate, pyruvate and acetate), were significantly upregulated. These pathways feed into the citric acid cycle and are essential for maintaining the NAD⁺/NADH ratio for cellular redox balance. GO terms related to the mitochondrial respiratory chain, particularly Complex I (Group 4), show mixed signatures in *Pyroxd1* KO myoblasts—though targeted assessment of the most profoundly affected proteins within these GO clusters supports overall adaptive upregulation in response to *Pyroxd1* KO (Supplementary Fig. 11C-F). Group 2 proteins, involved in endocytosis, vesicular trafficking, and proteasome function, were also upregulated in KO myoblasts. Acute loss of Pyroxd1 attenuated the thapsigargin-induced unfolded protein response (UPR) signature (Group 1, KO vs WT + thaps), consistent with impaired *XBP1* splicing (Fig. 3E). However, PCA shows that *Pyroxd1* expression status still accounts for a greater proportion (53%) of variance between samples than thapsigargin treatment (28%) (Supplementary Fig. 11A).

In *Pyroxd1* KO skeletal muscle, proteomics analyses revealed widespread mitochondrial dysfunction, with pronounced downregulation of proteins involved in mitochondrial function, particularly beta-oxidation and Complex 1 (Fig. 8C and Supplementary Fig. 12C-E). This inverse mitochondrial response, upregulation in KO cells and down regulation in KO muscle, may reflect differences between an acute cellular response and tissue-level adaptations in vivo. Collectively, our findings implicate Pyroxd1 as a critical upstream regulator of multiple essential pathways, with its depletion triggering acute mitochondrial dysfunction, impaired cell division (Fig. 3D), an attenuated unfolded protein response and eventual cell death.

PCA shows *Pyroxd1*_N155S_ skeletal muscle replicates cluster separately to samples from *Pyroxd1*_WT_, *Pyroxd1*_N155S/WT_ and *Pyroxd1*_N155G_ mice (Supplementary Fig. 13A), consistent with a benign (or minimal) phenotype in heterozygous or *Pyroxd1*_N155G_ mice. Therefore, further proteomic comparisons focused upon *Pyroxd1*_N155S_ versus *Pyroxd1*_WT_ mice (Fig. 8D), with additional comparisons of other benign genotypes presented in Supplementary Fig. 13. GO pathway analysis revealed significant upregulation of tRNA ligase complex components in *Pyroxd1*_N155S_ mouse muscle, consistent with its known role in protecting the tRNA ligase from oxidation-induced impairment. Notably, numerous proteins involved in pre-messenger RNA splicing were significantly downregulated, particularly those associated with catalytic step 2 (exon ligation), a process distinct from the function of the tRNA ligase complex (Fig. 8D and Supplementary Fig. 13). The most significantly downregulated proteins in *Pyroxd1*_N155S_ muscle were Acta2, AOX1, Atp2a3, ABCA12, Amd2, Myh8, Bpnt2 and Phkb, which are involved in muscle structure, metabolism, and calcium handling. Conversely, the most upregulated proteins in Pyroxd1_N155S_ muscle were Cpne5, Mlf2, Ablim1, Hspb7, Tceal7, and Xirp2, associated with transcriptional regulation, cytoskeletal dynamics, and stress responses (Fig. 8Dii).

### Protein modelling supports benign effects of N155G.

**Fig. 9:**
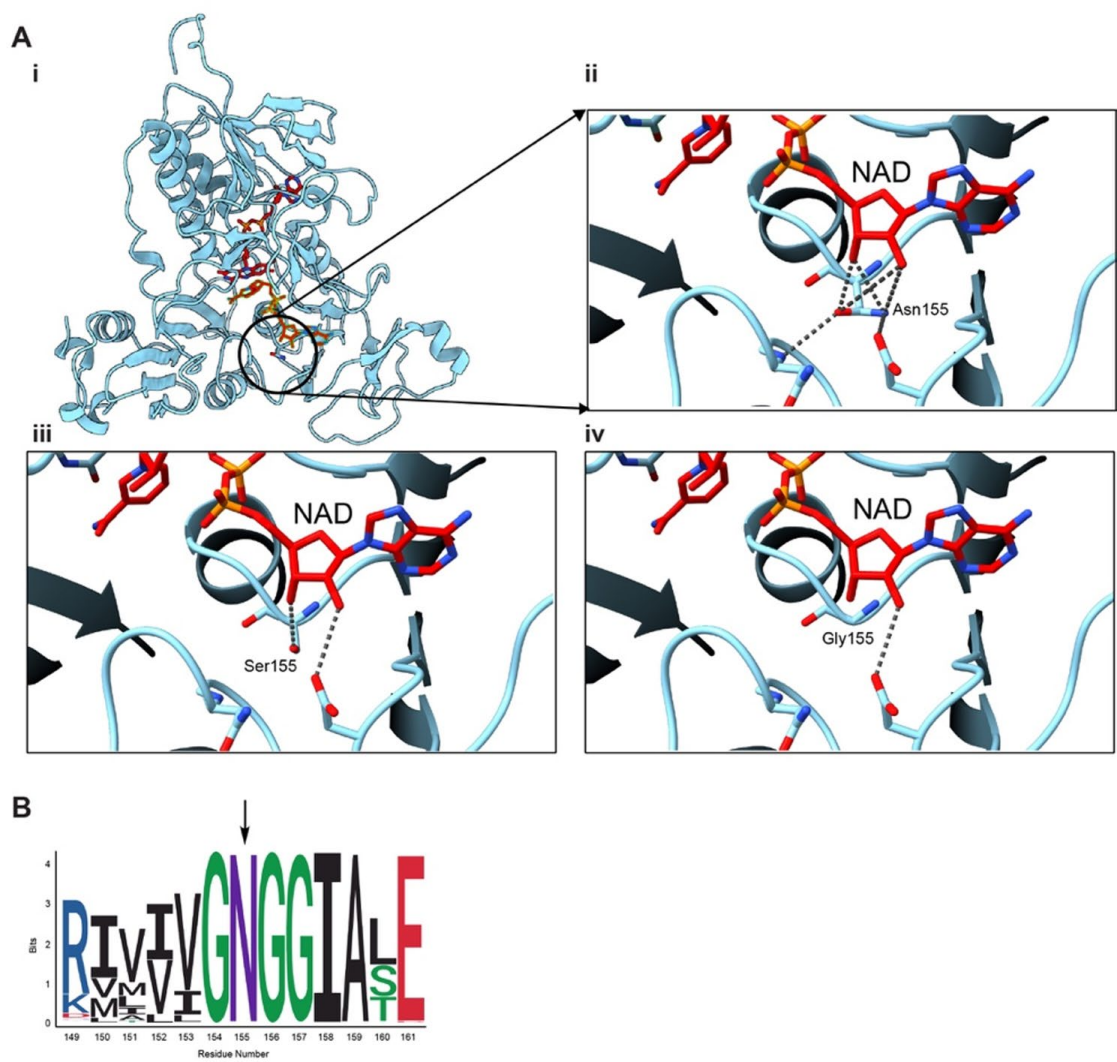
Protein modelling of amino acids at position 155. **A** i PYROXD1 structural model generated based on the cryo-EM structure of the PYROXD1–human tRNA ligase RTCB complex (PDB ID: 8ORJ [14]). Missing regions in 8ORJ (residues 1–10, 44–74, 203–231, and 286–293) were rebuilt using Modeller [24] with the human PYROXD1 amino acid sequence (UniProt ID: Q8WU10) as the reference. ii Native Asn155, iii Ser 155 substitution, iv Gly155 substitution. Dashed lines represent static hydrogen bonding distances. All lines are < 3.5 Å in length. **B** Logo plot was calculated from a ClustalO alignment of a dataset of 2364 protein sequences annotated as PYROXD1 from the NCBI protein database

Our studies support *Pyroxd1*_N155G_ mice being indistinguishable from *Pyroxd1*_WT_ mice, with no observable phenotype, despite the high evolutionary conservation of PYROXD1 Asn155 from unicellular organisms through to humans [3]. Structurally, Asn155 forms three strong hydrogen bonds with the adenosine ribose of the NAD moiety, along with secondary interactions with nearby residues and the protein backbone making Asn155 critical for maintaining a high-affinity NAD binding site (Fig. 9). Substituting Asn155 with Gly155 eliminates potential side chain interactions but the added loop flexibility may allow preservation of key hydrogen bonding interactions with NAD. Protein modelling suggests Gly155 can maintain, or even improve NADH binding compared to Asn155, consistent with the presence of a glycine residue at this position in other flavoproteins where it supports NADH coordination (Fig. 9). In contrast, structural modelling indicates that while the Ser155 backbone retains the same relative rigidity as the Asn155, its side chain cannot form hydrogen bonds with the NADH cofactor or neighbouring residues (Fig. 9).

## Discussion

We present a suite of mouse models designed to elucidate the function(s) of PYROXD1 and the underlying pathogenic basis for *PYROXD1* myopathy while also providing a future model system for pre-clinical therapy development. This includes a homozygous *Pyroxd1*_N155S_ mouse model of the recurrent human variant, which phenocopies key features of a severe form of human *PYROXD1* disorders. Our work highlights important distinctions between complete loss of Pyroxd1 (KO) and expression of dysfunctional Pyroxd1_N155S_. Early embryonic lethality of a global *Pyroxd1* KO mouse (between ED4.5–9.5), combined with impaired cell division following stage-specific *Pyroxd1* knockout, reinforce a vital role for Pyroxd1 in both cellular and organismal viability that cannot be substituted by any other oxidoreductase. Glutathione reductase and thioredoxin reductase were not measurably affected by Pyroxd1 deficiency or dysfunction in our murine or cell models.

We determined the protein half-life of Pyroxd1 to be ~ 19 h in cultured mouse cells, relevant for therapy development for *PYROXD1* disorders. While cell division in culture and early embryonic development may introduce dilutive effects, the in vivo defined window of KO embryonic lethality (within 4.5–9.5 days) is broadly consistent with our in vitro determined half-life. Although inducible *Pyroxd1* KO cell lines do not recapitulate the context of *PYROXD1* myopathy, they provide a powerful tool to study the fundamental biology of PYROXD1.

Embryonic lethality of global Pyroxd1 KO mice between ED4.5–9.5 aligns with key developmental stages providing potential insight into Pyroxd1 function. During embryo gastrulation (ED6.25–9.5) changes to glucose metabolism dictate cell differentiation [25] and there are significant changes in mitochondrial function immediately post-implantation (ED4.5) [26]. Other related redox enzyme KO mouse models have variable phenotypes; glutathione reductase KO mice are viable [27], while complete KO of thioredoxin reductase [28] or dihydrolipoamide dehydrogenase (*Dld*) [29] is murine embryonic lethal highlighting common essentiality of particular redox enzymes.

Expression studies in LacZ reporter and WT mice, and human muscle and myotubes, demonstrate *PYROXD1* is expressed at low levels at all stages of life, thus the mechanistic basis for the predominant skeletal muscle and connective tissue involvement in PYROXD1 disorders remains unclear. We postulate the unique oxidising environment of skeletal muscle, combined with its high metabolic demands for ATP to power ion pumps for excitation–contraction (EC) coupling, and intense need for fibrillar protein biosynthesis, may render it particularly vulnerable to reduced PYROXD1 activity.

*Pyroxd1*_N155S_ mice recapitulate key features of human PYROXD1 myopathy, representing the first validated murine model to study disease mechanism and pre-clinical therapy testing. Premature death of male *Pyroxd1*_N155S_ mice is a murine specific complication linked to species-specific anatomy [20] and has not been reported in humans. While this limits the use of male *Pyroxd1*_N155S_ mice in longitudinal studies, it does not reflect a human relevant aspect of PYROXD1 pathology. Skeletal muscle histopathology (internal nuclei, patchy mitochondrial staining and nemaline rods) in *Pyroxd1*_N155S_ mice mirrors that seen in human *PYROXD1* myopathy [3], but surprisingly the compound heterozygous *Pyroxd1*_N155S/KO_ mouse was embryonic lethal. While individuals carrying one null allele in trans with c.464A > G;p.(N155S) typically present with a more severe disease course than those homozygous for the N155S variant [7], this variant combination is viable in humans. However, it is possible that other variant combinations are not detected in the human population due to early lethality. The lethal *Pyroxd1*_N155S/KO_ murine phenotype may reflect murine-specific differences in skeletal muscle composition and function, or may reflect a particular consequence of this this specific variant combination.

Interestingly, homozygous *Pyroxd1*_N155G_ mice exhibit minimal detectable phenotypic anomalies. This finding is important for *PYROXD1* variant curation and is supported by protein modelling that suggests a glycine substitution at position 155 is likely to retain NADH binding activity and preserve PYROXD1 enzymatic function.

Muscles from *Pyroxd1*_N155S_ mice exhibit a notable transition of type 2X to 2B fibres of reduced fibre diameter, with relative preservation of type 1 and 2A fibres. This pattern deviates from the fast-to-slow fibre adaptive response seen in other myopathies due to variants affecting fast fibre gene products [30, 31]. Although both 2X and 2B murine skeletal muscle fibres are classed as fast-twitch glycolytic fibres, 2X fibres have twitch properties and fatigue resistance intermediate between 2A and 2B fibers [32]. *Pyroxd1*_*N155S*_ quadriceps muscles, composed of fast-twitch fibres, show widespread sarcomeric disruption on electron microscopy, patchy mitochondrial activity stains on histology and EDL muscles with similar fast-twitch properties showed reduced contractile force via ex vivo physiology. Conversely, *Pyroxd1*_N155S_ soleus muscle, composed of slower, oxidative type 1 and 2A fibres, has mild histopathological features and normal contractile force. Taken together, our findings indicate that Pyroxd1 dysfunction affects fast glycolytic muscle fibres more profoundly than slower oxidative muscle fibres.

Accounting for their reduced size, *Pyroxd1*_N155S_ muscles exhibit a clear force deficit using both in vivo and ex vivo force measurements, in line with significant observed sarcomeric disruption on electron microscopy. Interestingly, *Pyroxd1*_N155S_ muscles showed no significant deficits in force-frequency (EC coupling), passive force (mostly titin, connective tissue and possibly intermediate filament mediated), active force (actin-myosin cross-bridging) and eccentric contraction induced injury. Fatigue is a reversible loss of muscle function from strenuous contractile activity and is linked to reduced cross-bridge cycling and EC-coupling due to metabolite accumulation, altered intracellular Ca^2+^ handling and changes in membrane potential [33, 34]. Given the mitochondrial pathology and mitochondrial proteomic changes in *Pyroxd1*_N155S_ muscles, we anticipated increased fatigability. The unexpected fatigue resistance may be explained by improved oxygenation of the smaller *Pyroxd1*_N155S_ muscles during ex vivo testing, an increased proportion of strongly bound cross bridges demonstrated by active stiffness measurements and as an overall consequence of reduced force production due to having fewer functional sarcomeres. Based on published diffusion modelling [35] our experimental conditions (duty cycle = 0.5, temp = 22°C) yield an O_2_ diffusion radius of ~ 0.2 mm. With an average cross-sectional radius of ~ 0.47 mm for WT versus 0.34 mm for *Pyroxd1*_N155S_ muscles, this likely results in a larger hypoxic core in isolated *Pyroxd1*_WT_ skeletal muscles.

The bone phenotype seen in *Pyroxd1*_N155S_ mice mirrors the osteopenia recently described in humans with PYROXD1 myoathy [7]. Despite significant cortical and trabecular thinning, features that usually result in breakages in mouse models of osteogenesis imperfecta (OI) [36], no spontaneous fractures were detected in *Pyroxd1*_N155S_ mice. While reduced mechanical loading from significant skeletal muscle atrophy may contribute to the bone phenotype, tibia length and organ size was normal, excluding a skeletal or global growth defect. Previous studies have shown some bone abnormalities in the *mdx* mouse model of Duchenne muscular dystrophy [37, 38] but there is no available literature on bone phenotype in other mouse myopathy models.

Electron microscopy of *Pyroxd1*_N155S_ mice skin and aorta did not reveal a structural abnormality of collagen fibrils. Collective findings suggest that the osteopenia in *Pyroxd1*_N155S_ mice may stem from a bone-specific insufficiency of collagen production, transport and/or remodelling of existing fibrils, rather than the incorporation of structurally defective collagen. Bone and skeletal muscle, tissues that appear most vulnerable to PYROXD1 dysfunction, are notable for their abundance of fibrillar proteins. As PYROXD1 plays a key role maintaining tRNA ligase complex function [1, 14], we considered a potential common “supply chain” issue arising from reduced availability of mature intron-containing tRNAs that encode hydrophobic amino acids such as tyrosine, leucine, isoleucine, phenylalanine, and tryptophan. These residues are enriched in highly abundant skeletal muscle structural proteins myosin and actin, but not in collagen, which is more abundant in glycine, proline, and hydroxyproline. While a chronic disruption in the synthesis, transport or remodelling of fibrillar proteins provides a plausible explanation for the vulnerability of bone and muscle to the long-term effects of Pyroxd1 dysfunction, we do not yet have a definitive link between the muscle and bone phenotype observed here and tRNA ligase complex function. Notably, no overt amino acid deficiencies are detected in serum or urine from *PYROXD1* myopathy patients [7].

Proteomics analyses reinforce the phenotypic observations in our mouse models and previous studies implicating roles for Pyroxd1 in regulation of the tRNA ligase complex [1] and mitochondrial function [2]. We observe key differences between proteomic signatures in skeletal muscle with acute Pyroxd1 KO versus long-term adaptive responses of expression of *Pyroxd1*_N155S_. The opposing responses of mitochondrial and metabolic pathway effectors to Pyroxd1 KO with upregulation in cultured myoblasts versus downregulation in skeletal muscle may reflect differences in redox distress mitigation strategies. Cultured cells are bathed in media with abundant metabolite availability and high antioxidant buffering capacity, whereas intact tissue in vivo is subject to biological and biophysical constraints, including diffusion gradients and reliance on active transport mechanisms for metabolite uptake and by-product removal.

Proteomic analyses suggest a novel role for Pyroxd1 in the second catalytic step of pre-mRNA splicing, exon ligation. The rapid demise of *Pyroxd1* KO cells and embryos cannot be attributed to lack of tRNAs alone, given the long half-life of mature tRNAs, in the order of 100 hours [39]. However, a role for Pyroxd1 in pre-mRNA splicing in addition to tRNA ligase complex regulation could result in a combined shortage of mature mRNA transcripts *and* key tRNAs, potentially accounting for the eventual lethality of Pyroxd1 KO models. Taken together with the profound disruption of mitochondrial respiratory chain activity this may offer a plausible explanation for the acute lethality associated with Pyroxd1 KO. While previous studies support a role for Pyroxd1 in mitochondrial respiration [2] and regulation of the tRNA ligase complex [1, 14], we acknowledge that further functional validation assessing the role of Pyroxd1 in pre-mRNA splicing is required.

PYROXD1 is known to protect RTCB (RNA-splicing ligase RtcB homolog), the catalytic subunit of the tRNA ligase complex, from oxidative inactivation by shielding its active site from reactive oxygen species and Cu^2+^, thus preventing oxidation of the RTCB active-site cysteine residue [14]. It is therefore possible that PYROXD1 performs a similar redox protective function for other Cu^2+^ containing enzyme complexes, such as the mitochondrial respiratory chain complex I. Despite this possible link, we have no observed evidence for mitochondrial localisation of endogenous or transfected forms of PYROXD1 [3] (and unpublished data). While a mitochondrial localisation of PYROXD1 cannot be ruled out, our current evidence more strongly supports a nuclear or cytoplasmic role for PYROXD1 that indirectly regulates mitochondrial function, particularly complex I, perhaps through regulation of a critical co-factor or interacting enzyme.

In conclusion, our suite of extensively characterised murine models and temporally controlled *Pyroxd1* KO cell lines reveal multifaceted, essential cellular roles for PYROXD1. Key pathways affected include protein biosynthesis and turnover, tRNA ligase complex activity and the unfolded protein response, and mitochondrial respiratory chain function, with an implicated broader role in the exon-ligation step of pre-mRNA splicing. Our models provide both a foundational platform for mechanistic discovery and for future therapeutic development, supported by clear and quantifiable biomarkers to assess therapy response. Collectively, our research supports that PYROXD1 disease is caused by loss of PYROXD1 function, supporting PYROXD1 replacement as a potential therapeutic approach. As a therapeutic target, *PYROXD1* disorders have an advantage in that all affected individuals retain some residual PYROXD1 activity, reducing the risk of immune rejection replacing wild-type PYROXD1 protein. Moreover, the low endogenous levels of PYROXD1 across tissues suggests that even a modest restoration of wild-type PYROXD1 may provide substantial clinical benefit.

While the precise pathomechanism(s) of *PYROXD1* disorders remain to be fully delineated, our findings point toward a fundamental failure in cellular maintenance of redox balance, affecting numerous critical biosynthetic pathways. Our murine and cell models may accelerate therapeutic development and understanding of PYROXD1 as a master-switch regulating multiple essential redox pathways, as PYROXD1 supplementation therapies may have clinical application for other conditions associated with redox imbalance such as neurodegenerative disorders.

## Methods

### Mouse maintenance

All mice were maintained on a 12-h light–dark cycle with constant access to standard chow and water. Mouse phenotype was closely monitored in accordance with animal ethics regulations (Kids Research Transgenic Facility: K380, Westmead Institute for Medical Research: 4364, Australian Bioresources: 21/22, Murdoch Children’s Research Institute: A969) and animals sacrificed if adverse health events were observed. Genotype of mice was monitored during breeding by tail or ear clip DNA analysis.

Mice at experimental endpoints were sacrificed by cervical dislocation and collected tissues were frozen by submerging in partially-melted 2-methyl-butane for 30 s before transfer to dry ice and storage.

### Mouse embryo LacZ staining

X-gal staining was performed and imaged as previously described [40].

### Inducible Pyroxd1 KO mice

The PFLP-UBC mouse line is an inducible *Pyroxd1* KO model in which exon 5 of *Pyroxd1* is flanked by *LoxP* and Cre-ERT2 cassette is integrated into the genome. Upon tamoxifen injection, Cre-ERT2 translocates to the nucleus, excises Exon 5 by cleaving the *LoxP* sequences. This induces a frame-shift which results in a premature stop codon. Resulting *Pyroxd1* mRNA transcripts are targeted by nonsense mediated decay induced degradation producing a functional *Pyroxd1* KO. A small percentage of progeny from the PFLP-UBC mouse line were found to have undergone a recombination event before tamoxifen treatment. 

### Tamoxifen injections to induce murine Pyroxd1 KO

3-week-old PFLP-UBC mice were given intraperitoneal saline control or tamoxifen injections on 2 consecutive days to induce *Pyroxd1* KO. Tamoxifen was prepared in corn oil (Sigma), administered at 375 ng/g dosage, and mice were weight and health monitored twice daily for the duration of the trial. Following 2 × IP tamoxifen injections on consecutive days mice were sacrificed, at 36 h, 5 days, and 7 days post final injection and tissue collected and frozen for all downstream analysis.

### Generation of the Pyroxd1_N155S_ and Pyroxd1_N155S/KO_ mouse

Mouse line creation was performed by Australian BioResources (ABR) MEGA service using CRISPR-Cas9 base-editing on a C57BL/6 background. Asn155 (AAT) was edited successfully to Ser155 (AGT), enabling derivation of the homozygous *Pyroxd1*_N155S/N155S_ line. Editing also incidentally created a line carrying an Asn155 (AAT) to Gly155 (GGT) substitution, Pyrod1N155G. Mice homozygous and heterozygous for the N155S mutation are referred to as *Pyroxd1*_N155S_ and *Pyroxd1*_WT/N155S_ (or S/S and S/WT) respectively, while those homozygous for the N155G mutation are referred to as *Pyroxd1*_N155G_ (or G/G). To create a compound heterozygous *Pyroxd1*_N155S/KO_, we bred heterozygous *Pyroxd1*_N155S/WT_ mice with heterozygous *Pyroxd1*_KO/WT_ mice and genotyped offspring.

### WGS of Pyroxd1_N155S_ mice to assess potential off target effects of base editing

WGS was performed by Australian Genome Research Facility (AGRF), DNA Prep PCR-Free with NovaSeq 150 bp paired-end reads. For analysis, VCF files for WT (BL6), homozygous *Pyroxd1*_N155S_ and homozygous *Pyroxd1*_N155G_ mice (with pre-existing Ensembl VEP annotations) were merged into a single multi-sample VCF file using bcftools. Variant quality metrics were inspected using R. Variants confidence was subsequently assessed using GATK Variant Recalibrator using a set of high confidence variants source from combined WT(BL6), *Pyroxd1*_N155G_ and *Pyroxd1*_N155S_ VCF and Wellcome Sanger Institute Mouse genome project.

Combined VCF for WT(BL6), *Pyroxd1*_N155G_ and *Pyroxd1*_N155S_ were run through ApplyVQSR to identify high confidence SNPs and Indels. Variants were further annotated identify if they (a) Resided within Ensembl Release 102 protein coding genes; (b) Identified mouse gene was associated with murine lethality as identified by IMPC; (c) Mouse gene's human ortholog was associated with OMIM phenotype.

The following steps were taken to identify potential variants:

- 307,581 variants were segregated into Exome (88,562) / Intergenic (219,019)
- Exome variants were filtered down to those passing VQSR filters (87,996)
- Hard filtering was applied to remove Genotype for variants with GQ < 20
- Variants with 3 or more missing genotypes (across the six BL6, NG and NS samples) were excluded from further analysis (26,888), leaving 61,108 for further analysis
- Variants with splicing, missense or stop start/gain (as per VEP) and where the gene was associated with murine lethality/Human ortholog OMIM phenotype were identified for further analysis (222)
- Finally, out of 222, variants with differing genotypes across all the samples and not annotated in dbSNP 150 were selected for manual curation (62)

### Cell culture

Myoblast cell lines were derived via explant culture from relevant mouse colonies as previously described [41]. Cells were maintained in DMEM:F12 (Life Technologies) supplemented with 20% heat-inactivated fetal bovine serum (GE HyClone), 5% Amniomax II complete medium (Life Technologies) and 50 μg/ml gentamicin (Life Technologies). Pyroxd1 knock out was induced by incubation with 2 µM 4-hydroxytamoxifen (Sigma Aldrich) for relevant times.

### Human muscle biopsies

Human muscle biopsies were obtained under Ethics approval by the Children’s Hospital at Westmead Human Research Ethics Committee (Biospecimen Bank_10/CHW/ 45 and 2019/ETH11736) with informed, written consent.

### Western blot

Cell pellets and tissues were solubilised, protein concentration determined, and western blotting performed as previously described [42] with loading according to individual experiments as indicated in figure legends.

Antibodies used were against PYROXD1 (Abcam ab122458, now discontinued), PYROXD1 (custom made polyclonal, kind gift of Professor Javier Martinez, MFPL and MedUni Wien), GAPDH (MAB374, Merck Millipore), β-tubulin (E7, AB_2315513 Developmental Studies Hybridoma Bank, Iowa) and Glutathione Reductase (ab16801, Abcam).

### Pyroxd1 protein half life

Cre + PFLP-UBC cells were treated with vehicle DMSO or 4OH-Tamoxifen (Sigma) to induce *Pyroxd1* recombination and functional protein knockout. PCR was used to confirm the timing of the recombination event (signalling the start of protein exponential decay), and western blot performed to determine Pyroxd1 protein levels over four days. Using results from four replicate derived inducible Pyroxd1 KO myoblast cell lines, Pyroxd1 protein relative intensities were plotted over the time course of the experiment and its half-life was calculated.

### Flow cytometry cell trace violet proliferation assay

Cre + mouse myoblasts were cultured in a proliferating undifferentiated state in complete culture medium containing 4-OHT (KO) or DMSO (WT). After 4 days, cells were trypsinised, pelleted, and resuspended in CellTrace Violet (ThermoFisher Scientific) diluted 1:1000 in warm DPBS and incubated at 37 °C for 20 min. Warm complete media was then added to the cells, and incubated 37 °C for 5 min. 1 × 10^5 from each sample were taken for flow cytometry analysis, and the remaining cells were pelleted, resuspended in complete media, and seeded into a 6-well plate. For flow cytometry, cells were pelleted, washed once in DPBS, resuspended in DPBS with 1:100 7-AAD (BD Biosciences), and passed through a cell strainer (Falcon). Analysis of the CellTrace Violet signal was carried out on one well of cells for each treatment group every 24 h until day 7. The median fluorescence intensity of the 7-AAD-negative population was normalised to that of day 4 for each treatment group.

### Mouse genotyping PCRs

Genomic DNA was extracted from cells and tissues using a GenElute Mammalian genomic DNA Extraction miniprep kit (Sigma) according to manufacturer’s specifications.

The PFLP-UBC mouse line recombination events were detected using primers KO-F 5’-CGTTCCCAAAGGCGCATAA-3’ and KO-R 5’-AACACACACCCTAACCAGCA-3’ which overlap the *LoxP* sequences flanking *Pyroxd1* exon 5 and produce an unrecombined 980 bp or recombined 220 bp product. Cycling conditions were: 95 °C for 3 min, 40 cycles of (95 °C for 20 s, 61 °C for 20 s, 72 °C for 1 min 10 s), and 72 °C for 7 min final extension. Inheritance of the Cre-ERT2 transgene was detected using primers Cre-AF 5’-CTGACCGTACACCAAAATTTGCCTG-3’ and Cre-AR 5’-CACCAGCTTGCATGATCTCC-3’ with cycling conditions 95 °C for 3 min, 40 cycles of (95 °C for 20 s, 58 °C for 20 s, 72 °C for 1 min 10 s), 72 °C for 7 min final extension. Alternatively, a multiplex PCR was used with primers Cre-F 5’-CATCGTCGGTCCGGGCTGCC-3’, Cre-R 5’-CCCCCAGGCTAAGTGCCTTC-3’, Pyroxd1NTerm-F 5’-TAACAGCCGGAAAGTCTGCC-3’, Pyroxd1NTerm-R 5’-TGTCTACTCCTGGCCCCAAA-3’ with cycling conditions 95 °C for 3 min, 30 cycles of (95 °C for 20 s, 61 °C for 20 s, 72 °C for 30 s), 72 °C for 7 min final extension.

Genotyping *Pyroxd1*_N155S_ and *Pyroxd1*_N155G_ mouse lines was achieved by using discriminating forward primers with either a single 3’ end base mismatch to WT sequence (detecting the WT allele) or two end base mismatches (detecting both N155S and N155G mutations). The combination of two PCRs with WT forward primer 5’-GCAAAAGCTAGGAGGATAATGATTGTCGGGCA-3’ or mutant forward primer 5’-GCAAAAGCTAGGAGGATAATGATTGTCGGGCG-3’ with common reverse primer 5’-CACATTCTGGACCTGCCATGTTTAGGGAGA-3’ was used to discriminate zygosity. Cycling conditions: 95 °C for 3 min, 40 cycles of (95 °C for 15 s, 68 °C for 30 s), and 72 °C for 5 min.

### *XBP1* RT-PCR

RNA was extracted as previously described [42]. To interrogate the functionality of the unfolded protein response *XBP1* splicing was assessed using RT-PCR as previously described [7, 43, 44].

#### Skeletal muscle histology

Frozen quadriceps muscle sections  (8µm thick) were collected on SuperFrost plus slides and used for histological assessment.

*Wheat Germ Agglutinin/DAPI and Hematoxylin and Eosin (H&E) Staining* was used to visualise overall muscle architecture, and visualise and count internalised nuclei as described in [45].

*Nicotinamide adenine dinucleotide (NADH) dye staining:* Used to visualise mitochondrial integrity in frozen quadriceps muscle sections. NADH dye solution dissolved in 0.05M Tris buffer pH 7.6 (1.6mg/ml) was mixed 1:1 with Nitro blue tetrazolium (NBT) also dissolved in 0.05M Tris buffer pH 7.6 (2mg/ml) and applied to thawed muscle sections for 30 min at 37°C in a humidifying chamber. Slides were washed 3 times in deionised H_2_O then immersed 3 × in increasing then decreasing concentrations of 30, 60 and 90% acetone. Slides were left in 90% acetone until a faint purplish cloud was seen over the section. Finally slides were washed several times in deionised H_2_O before being mounted with Immu-Mount™ (9990402, Epredia™) and coverslipped.

*Alcian Blue/Picosirius Red Staining*: Used to visualise cartilage and collagen content in decalcified paraffin bone sections. Slides were dewaxed in xylene (2 × for 5 min), 100% ethanol (2 × for 1 min), 70% ethanol (30 s) then rinsed in deionised H_2_O. Slides were stained for 15 min with Weigert’s iron hematoxylin (115973, Merck Millipore). Sections were washed under running tap water for 5 min and the stained with Alcian Blue (1% in 3% acetic acid, pH 2.5) for 10 min. Sections were rinsed with distilled water, stained with 1% aqueous phosphomolybdic acid for 2 min, rinsed with distilled water before staining for 90 min with 0.1% Sirius Red diluted in concentrated Picric acid. Sections were then dehydrated for 3 min each in 70%, 95% and 100% ethanol (2x) and xylene (3x). Slides were finally mounted in Eukitt mounting media (03989, Sigma Aldrich).

*Fiber typing:* Sections were briefly thawed and serum blocked in 2% BSA/PBS for 15 min, then blocked for 1h with fab fragment goat anti-mouse IgG (1:50, 115-007-003, Jackson ImmunoResearch) prior to overnight incubation at 4°C in anti-MyHC 2B (1:40, BF-F3, mouse IgM, University of Iowa) and polyclonal rabbit anti-dystrophin (1:400, ab15277, Abcam). On the next day, slides were washed 3 × in PBS and then blocked again in 2% BSA for 15 min, prior to addition of secondary antibodies 1 in 200 goat anti-mouse AF-555 IgM (A-21426, Thermo Fisher Scientific) and 1 in 200 goat anti-rabbit AF647 IgG (A-21245, Thermo Fisher Scientific) incubated for 2 h at room temperature. The primary antibody for detection of type 1 (slow) fibres, MyHC 1 (MAB 1628, Chemicon) and primary for type 2A fibers, MyHC 2A (SC-71, University of Iowa) were labelled with AF-350 and AF-488 respectively using the Zenon labelling kit according to the manufacturer’s instructions (Thermo Fisher Scientific). Following secondary antibody incubation, sections were washed 3 × in PBS then incubated for 2h at R.T with Zenon labelled primary MyHC 2A and MyHC 1, each diluted 1 in 20. Slides were washed 3 × in PBS, fixed with 4% paraformaldehyde for 10 min, washed again 3 × in PBS before finally mounting with Immu-Mount™ (9990402, Epredia™). Imaging was performed on the Leica DMi8 Inverted Microscope (Leica Microsystems). Images were then analysed for fibre size and type using a custom-made Image J macro. Data output from Image J was further analysed in Excel and Graphpad Prism.

### Electron microscopy and quantification

Following harvest, tissues for electron microscopy were placed directly in Karnovsky's fixative. Following Karnovskys fixative overnight, samples were washed in MOPS buffer. Post fixation in 2% osmium tetroxide was then followed by dehydration in ethanol at concentrations of 50%, 70%, 95% and 100% for 15 min each. Dehydration was continued with 100% dry acetone for 20 min. Blocks were infiltrated with acetone/ TAAB Low Viscosity Resin (TAAB Laboratories) in a 1:1 ratio for one hour and then further infiltrated with three changes of 100% resin for 15 min each at 70 °C. Blocks were embedded in resin and polymerized overnight at 70 °C.

Sections were cut at 90 nm using a UC6 ultramicrotome (Leica Microsystems), mounted on grids and stained with 2% uranyl acetate in 50% ethanol (15 min) and Reynold’s lead citrate (4 min). Grids were examined with a PhilipsCM120 BioTwin transmission electron microscope (Westmead Scientific Platforms) operating at 100 kV,with images recorded using a Morada camera and iTEM software (Olym-pus SIS).

The diameter of Collagen fibres from EM images was quantified using a custom written Image J macro. Data was exported to excel and analysed using Graphpad Prism.

### Bone micro-CT

Whole mouse tibiae were removed, wrapped in gauze, and soaked in saline before freezing at -80 °C for storage. Scans were conducted using the SkyScan1272 (Bruker, Kintich, Belgium) with parameters as previously described [46]. Separate trabecular and cortical regions of interest were delineated based on offset distance from inferior end of the metaphyseal growth plate. Standard regions of interest [46] for cortical (1.7 mm offset, 1.2 mm height) and trabecular (5.5 mm offset, 0.5 mm height) bone were delineated using NRecon software (Bruker). Cortical bone thickness (CtTh), bone volume (CtBV), mean polar moment of inertia (MMI) and trabecular bone mineral density (BMD), bone volume (TbBV), thickness (TbTh), separation (TbSp), and number (TbN) were reported by CTAn analysis software (Bruker).

### Bone histology

Bones were prepared for histology as previously described [46]. Briefly, whole tibiae from 3-month-old male *Pyroxd1*_N155S_, *Pyroxd1*_WT/N155S_, *Pyroxd1*_WT_, and *Pyroxd1*_N155G_ mice were submerged in in situ decalcification solution at room temperature for 4 days. Tibiae were then transferred to standard decalcification solution for 7 days (changed after 3 days). Samples were then washed in 70% ethanol before being embedded in paraffin wax.

### Fore limb grip strength testing

Forelimb grip strength was assessed using a digital grip strength meter (Columbus Instruments, USA) as previously described [47]. Briefly, mice were held by the tail and allowed to grasp a horizontal metal hanger with their forepaws before being gently pulled back until the grip was released. The peak force achieved (in Newtons (N)) was automatically recorded by the transducer. Each animal performed five consecutive trials separated by ≥ 1 min rest to avoid fatigue, and the highest value was taken as the maximal grip strength. Grip strength was measured on the morning of functional experiments and values were normalized to body mass to account for inter-animal variability. All measurements were performed by the same investigator.

### Hindlimb intact muscle mechanics

Ex vivo contractile function of the extensor digitorum longus (EDL; fast-twitch) and soleus (SOL; slow-twitch) muscles was examined using established protocols [48–50]. Following euthanasia, muscles were carefully dissected and mounted by their tendons between a dual-mode force transducer/length controller (Aurora 1200A vertical preparation and Aurora 1300A horizontal preparations) in an organ bath containing continuously carbogenated (95% O₂/5% CO₂) Krebs solution (in mM: 118 NaCl, 4.75 KCl, 1.18 KH₂PO₄, 1.18 MgSO₄, 24.8 NaHCO₃, 2.5 CaCl₂, 10 glucose) maintained at 22 °C. Muscles were adjusted to optimal length (Lo) by eliciting maximal twitch contractions via supramaximal stimulation through parallel platinum electrodes.

Contractile protocols included the following (Note: left EDL/SOL underwent fatigue and recovery measures whereas right EDL/SOL underwent eccentric contraction measures):

Force–frequency relationship: trains of stimuli (2–150 Hz; 1 s for EDL, 2 s for SOL) with 30 s rest between contractions. A sigmoid curve relating the muscle force to stimulation frequency was fitted by linear regression to this data.

Passive stiffness: muscles were stretched by 5% Lo and the resulting passive force recorded.

Active stiffness and eccentric contractions: during a maximal tetanus, muscles were lengthened by 10% Lo at 1 Lo/s for 10 consecutive contractions, with 5 min rest between contractions.

Fatigue and recovery: a series of 10 maximal tetani (1 s on/1 s off for EDL; 2 s on/1 s off for SOL) was delivered until force declined to ~ 50% of initial. Recovery was monitored at set intervals up to 15 min.

At the conclusion of experiments, muscles were blotted dry and weighed. Physiological cross-sectional area (PCSA) was derived from muscle length, mass and skeletal muscle density as previously published [51]. Specific force was determined by normalizing Po to PCSA. All analyses were performed using Aurora DMC/DMA software.

### Mass spectrometry sample preparation

All samples were prepared for mass spectrometry proteomics analysis by lysing in 2% SDS, 50 mM TEAB and protease inhibitors and sonicating thoroughly. Samples were reduced with 10 mM tris(2-carboxyethyl)phosphine (TCEP) with incubation for 10 min at 85 °C and alkylated with 20 mM iodoacetamide for 30 min at 23 °C. The samples were precipitated using the chloroform–methanol method, then digested in 7.8 M urea with 100 mM 4-(2-hydroxyethyl)-1-piperazineethanesulfonic acid (HEPES) pH 8 and 2 μg of LysC (WAKO Fujifilm) at 28 °C for 8 h, followed by eight fold dilution with 100 mM HEPES and two rounds of digestion with 2 μg trypsin (Trypzean, MERCK) at 28 °C for 8 h. The remain steps were as described previously [52]. Briefly, the samples were quantified, labelled with TMTpro regents (Thermo Fisher Scientific), combined, desalted and the peptides applied to hydrophilic interaction liquid chromatography separation and fractions were collected. Each fraction was analysed using LC–MS/MS performed with a Dionex UltiMate 3000 RSLC nano system and Q Exactive Plus hybrid quadrupole-orbitrap mass spectrometer (Thermo Fisher Scientific). The LC–MS/MS was as described previously [52], except that the gradient was from 94% phase A (0.1% formic acid in water)/6% phase B (0.1% formic acid, 9.9% water, 90% acetonitrile) to 28% phase B in 71.5 min, then to 36% buffer B in 8 min. Data-dependent acquisition MS/MS acquisition was performed for 110 min. The MS/MS scans were for a maximum ion time of 115 ms, the normalised collision energy was 30 and dynamic exclusion was for 35 s.

### Proteomic analysis: differential abundance analysis of proteins

The raw MS data was searched using MaxQuant 1.6.7.0 [53]. Carbamidomethyl (Cys) was a fixed modification. Oxidation (Met), acetylation (N-terminal) and deamidation (Asn/Gln) were variable modifications. The enzyme specificity was trypsin/P with up to three missed cleavages. The TMTpro correction factors were entered (lot VB294905 for myoblasts and lot VJ313476 for muscle tissue samples). Minimum reporter peptide ion faction was 0.6. Minimum peptide length was 6 and maximum peptide mass was 6000 Da. The MS/MS spectra were searched against all entries within the Mus musculus UniProt reference proteome FASTA file downloaded Dec 29 2021 were used. All other parameters were set as the defaults in MaxQuant 1.6.7.0.

*Data Cleaning:* The “proteinGroups.txt” output file from MaxQuant were processed. Each protein group must have at least one unique peptide. A protein was removed from further analysis if the first accession in the protein group matches a protein contaminant or the sequences in the reverse sequence decoy databases, in which the protein accession starts with CON__ or REV__ prefixes respectively. The “reporter intensity corrected” column were used for further analysis. Proteins with one or more missing values in any samples were removed from further analysis. Proteins with any missing values were removed from the analysis.

Identifying a representative UniProt accession for each protein group: The following rules from Engholm-Keller et al [54]. were used to identify a representative UniProt accession for each protein group.

1. For proteins that mapped to multiple UniProtKB protein accessions, the accession with the lowest “protein existence (PE)” value was kept as the best evidence (http://www.uniprot.org/help/protein_existence). Where the protein accession was an isoform (therefore lacking PE information), the PE value was taken from the parent protein.
2. When the PE value was equal, a Swiss-Prot (sp) entry was taken over a TrEMBL (tr) entry.
3. If both entries were Swiss-Prot, the non-isoform was selected.
4. If both entries were isoforms, the longest isoform was selected.

*Data normalisation:* First, samples were log (base 2) transformed and between sample normalization were performed using the “scaled” normalization from the ‘limma’ R package [55]. Second, to remove the batch effects from biological data, the remove unwanted variation (ruv) R package [56] was used to remove batch effects. The method relies on having a set of endogenous negative control proteins, which are proteins with little changes in protein abundances between different cell types or experimental treatments. For this study, a set number of empirical negative control proteins listed in Table 1 with little or no change in protein expression across samples (q-value < 0.05) were identified from an initial ANOVA test. The RUVIII method [57] was used to remove the unwanted variations across the samples and the number of unwanted components listed in Table 1 were removed by the tool. The RUVIII method requires the experiment design matrix, a matrix describing the replicates for each treatment condition, and the list of negative control proteins. Differential abundance analysis of proteins was performed using the adjusted abundance matrix. The results from this differential abundance analysis were then used for all analyses.

**Table 1:** Statistical analysis parameters for each dataset

Dataset	Number of empirical negative control proteins	K	Log fold-change threshold for the treat function in R
N155S vs controls	100	2	NA
KO cells	300	2	1.5
KO muscle	100	1	NA

*Linear Modelling and pathways analysis:* Differential abundance analysis of proteins was performed for comparing each time point with time zero. The ‘limma’ R package [55] (Ritchie et al., 2015) was used, the linear model for comparing each pair of time points was fitted using the ‘lmFit’ function and the p-values calculated using the empirical Bayes method ‘eBayes’ function. Trended and robust analysis were enabled. The false discovery rate correction was applied to the moderated p-values by calculating the q-values (Storey, 2002). The treat function [58] was used to find proteins with high confidence log fold-change above the threshold. Significant differentially expressed proteins were defined as those with q-values less than 0.05. Pathways analysis was performed using the enricher function from the clusterProfiler R package [59].

*Determination of protein clusters for comparative heat map:* Clustering was performed using Euclidian distance and complete linkage clustering. The String DB functional enrichment tool [23] was used to identify the enriched GO terms for each cluster and the list of all proteins without missing values identified in the experiment was used as the background proteins list.

### Protein modelling

Structural analysis of the PYROXD1 protein and its variants was performed using ChimeraX. Mutations were introduced with the *swapaa* command, and interatomic distances were measured using the *distance* tool. Sequence logo analysis was conducted in R using the *ggseqlogo* package. A total of 2,364 sequences for the logoplot were obtained from a curated NCBI database of PYROXD1 orthologs and aligned using MUSCLE [60]. A custom Python script extracted a 13–amino acid window surrounding the selected motif for visualization.

## Supplementary Information

Below is the link to the electronic supplementary material.

Supplementary Material 1

## Data Availability

No datasets were generated or analysed during the current study.
