# Supplementary material for: Pyroxd1 is essential for murine viability with the homozygous N155S recurrent variant linked to myopathy, muscle hypotrophy and osteopenia
Source: Acta Neuropathol Commun. 2026 Mar 2;14:84. doi: 10.1186/s40478-026-02259-1 (PMC13059403; doi:10.1186/s40478-026-02259-1)
Supplement: Supplementary file 1 — Supplementary Material 1 [file 40478_2026_2259_MOESM1_ESM.docx]

Supplementary Figure 1

**Supplementary Figure 1. Schematics of reporter and KO mouse models.**

Supplementary Figure 2

**Supplementary Figure 2. Tamoxifen induced KO of PYROXD1 in PFLP-UBC mice varies across tissues and days post treatment.** A-F represent six individual mice, three in each condition. **A)** PFLP-UBC embryos treated with Tamoxifen on ED14-15 (through injection of the mother) and sacrificed on ED18 had genetic PYROXD1 recombination confirmed by PCR in Heart, Liver, and Brain. **B)** Western Blot was performed on these tissues with β-Tubulin or GAPDH as loading control. Half (½, lanes 4-5) and quarter (¼, lanes 6-7) loading of samples A and B demonstrate the antibody is in the linear range and reductions can be detected. rP (lane 11): purified recombinant human PYROXD1 protein. **C)** 4-week-old PFLP-UBC mice injected with Tamoxifen had genetic PYROXD1 recombination in skeletal muscle confirmed by PCR 36 hours, 5 days, and 7 days post treatment. Each sample represents an individual mouse from a terminal harvest. Asterisk (*) indicates a rare event where a Cre- mouse displayed Pyroxd1 recombination, likely due to aberrant Cre activity in utero. **D)** Western Blot was performed from each sample to detect PYROXD1 protein levels with total muscle content (Coomassie staining of actin) as a loading control.

Supplementary Figure 3

**Supplementary Figure 3**. **A)** Table summary of full report from Cerberus Sciences on histopathology from 16–17-week-old mice of submitted Kidney, Bladder, and Testes samples from mice of each genotype, and segregated by disease severity at time of sample fixation. Numbering indicates number of samples with each pathology found out of total samples in that group. Non-specific findings by Cerberus Sciences are omitted. **B)** Representative images from normal (*i*) and *Pyroxd1*_N155S_ bladders (*ii-iv*). Arrow heads mark walls of the lumen and exterior are normal in WT mice (i) while similarly arrow marked regions in sick *Pyroxd1*_N155S_ mice have neutrophil infiltration (*ii*). A proteinaceous urethral plug was observed in the bladder of the single dead *Pyroxd1*_N155S_ mouse analysed (*iii*) with a multifocal haemorrhaging in the bladder wall (*iv*). **C)** Renal tubules of *Pyroxd1*_WT_ (*i, ii*) and *Pyroxd1*_N155S_ (*iii, iv*) mice, with notable tubules indicated by arrows. **D)** Whole genome sequencing filtering as per methods to determine any genomic changes in *Pyroxd1*_N155S_ mice. ^1^Ensembl protein coding genes (Ensembl Release 102, GRCm38.p6). ^2^No genotype determined or low-quality genotype (Quality < 20) for 3 or more samples. ^3^Human ortholog genes with OMIM phenotype (Ensembl Release 102) / Mouse lethal genes from IMPC genotype phenotype Release 14.0 (where phenotype contains keyword ‘lethal’ or ‘death’) 3dbSNP 150 (Ensembl Release 102, GRCm38.p6).

Supplementary Figure 4

**Supplementary Figure 4. Gross morphology and weight measurements in each mouse genotype.**

**A)** Size comparisons of *Pyroxd1*_WT_ and *Pyroxd1*_N155S_ mice sacrificed at 4, 8, and 13 weeks. **B)** Images of mouse posture changes in 13-week-old *Pyroxd1*_N155S_ mice (arrow) compared to *Pyroxd1*_WT_ during rearing on hind limbs, and normal stance (not to scale). Total body **C)** and quadriceps muscle **E)** weights measured in mice sacrificed at 13 and 26 weeks. **D)** Size comparison of quadriceps and gastrocnemius muscles from 13-week-old male mice. Statistical significance is indicated by asterisk (*p<0.05, **p<0.01, ***p<0.001, ****p<0.0001). Abbreviations: WT: *Pyroxd1*_WT_, S/S: *Pyroxd1*_N155S_, G/G: Pyroxd1_N155G_, S/WT: Pyroxd1_WT/N155S_.

Supplementary Figure 5

**Supplementary Figure 5. Fibre type staining reveals presence of smaller more densely packed type 2B fibres in *Pyroxd1*_N155S_ mice**

**A)** Cross sectional area occupied by each fibre type across the entire muscle section expressed as a percentage of total area. This analysis was done on quadriceps muscle sections from 3-month-old mice. *Pyroxd1*_N155S_ (S/S, n=3) mice were compared to wildtype (WT, n=4), *Pyroxd1*_N155G_ (G/G, n=3) and *Pyroxd1*_N155S/WT_ (S/WT, n=3). **B)** Comparison of total number of fibres counted across the entire muscle section revealed no significant difference between the four genotypes. **C)** Comparison of muscle fibre density (MFD) expressed as total number of fibres divided by total cross-sectional area. An unpaired t-test between WT and S/S revealed a significant increase in MFD in S/S mice ***p=0.0009. Data in graph **B)** and **C)** is represented as mean ± SEM, each dot represents an individual animal. **D)** Additional whole muscle section fibre typing images showing differences in fibre type composition and staining intensity. One muscle WT sample which was stained like other three WT samples has been omitted for consistency of the image panel. Scale bar =500µm.

Supplementary Figure 6

**Supplementary Figure 6. Intact muscle mechanics cohort - hindlimb muscle weight and length**

Intact muscle mechanics was performed on 4-month-old WT (Pyroxd1_WT_), S/S (Pyroxd1_N155S_), G/G (Pyroxd1_N155G_), S/WT (Pyroxd1_WT/N155S_)_._ Soleus (SOL), extensor digitorum longus (EDL) and tibialis anterior (TA) muscle weight normalised to mouse body weight (BW) are shown in (A). (Ai) SOL weight/BW was not significantly different compared to WT in S/S, G/G and S/WT mice. (Aii) EDL weight/BW was significantly reduced in S/S mice (21.2% reduction; WT: 0.471 ± 0.027 n=15, S/S: 0.371 ± 0.045 n=13). TA weight/BW was significantly reduced in S/S mice and G/G mice compared to WT (34.5% and 10.8% reduction, respectively; WT: 2.293 ± 0.064 n=7, S/S: 1.502 ± 0.0999 n=7, G/G: 2.045 ± 0.177 n=10). (B) Optimal SOL (i) and EDL (ii) muscle length during ex vivo intact muscle mechanics measurements were not different between genotypes. Bars represent group mean and circles are individual mice. Values represent mean ± standard deviation. One-way ANOVA was used for statistical comparisons of WT to all other genotypes and significance is indicated (**** p >0.0001).

Supplementary Figure 7

**Supplementary Figure 7. Force comparisons between genotypes during fatigue protocol**

**A)** EDL force (% of original) following (*i*) 8 fatigue contraction, (*ii*) after final fatigue contraction (8th for WT, G/G and S/WT; 10th for S/S) and (*iii*) following recovery. **B)** SOL force (% original force) at the (*i*) final/26^th^ fatigue contraction and (*ii*) following recovery. Average fatigue end force loss was 46.8 - 49.7% in EDL and 54.3 - 63.7% in SOL.

Supplementary Figure 8

**Supplementary Figure 8. Passive stiffness**

**A)** EDL and **B)** SOL passive stiffness “In Series” and “In parallel”. Passive stiffness during a 5% stretch was determined using published methods. **C)** Example trace files showing a typical passive stretch protocol and how the stiffness data was derived.

Supplementary Figure 9

**Supplementary Figure 9. Eccentric contraction induced force loss is similar between WT and *Pyroxd1*_N155S_ mice**

Force loss during 10 eccentric contractions in **A)** EDL and **B)** SOL. A four-parameter logistic sigmoidal curve was fitted to the data and the mean ± standard deviation of the slope is shown in the bar graph inset. EDL end forces as % of initial: WT: 32.6 ± 6.24% (n=7), S/S: 31.0 ± 2.9 (n=6), S/WT: 19.1 ± 3.2 (n=3) and G/G: 27.5 ± 3.3 (n=10). SOL end forces as% of initial: WT: 78.0 ± 13.3% (n=6), S/S: 87.5 ± 5.5% (n=7), S/WT: 78.8 ± 9.0% (n=3) and G/G: 79.07 ± 9.1 (n=10)The hills co-efficient of S/S mice was increased compared to other groups in EDL muscle (*p=0.0353). **C)** Force and length trace of a typical muscle highlighting the isometric and eccentric part of the contraction. **D)** Using eccentric contraction data, we determined the fraction of active cross bridges (from active stiffness) during contraction in (i) EDL and (*ii*) SOL. We found that the amount of active cross bridges was significantly increased in the EDL of S/S mice compared to WT (WT: 57.0 ± 4.5 %, G/G: 55.2 ± 3.0 %, S/WT: 57.5 ± 2.1 %, S/S: 62.6 ± 5.8 %; **p=0.0087 one-way ANOVA, **D*i*)**. No differences were found in SOL **D*ii*)**.

Supplementary Figure 10

**Supplementary Figure 10.** Extended bone analysis and EM analysis of collagen fibrils from Pyroxd1_WT_ and Pyroxd1_N155S_ mice. **A)** Additional bone metrics comparing WT and Pyroxd1_N155S_ mice as well as Pyroxd1_N155G_ and Pyroxd1_N155S/WT_ mice at 13 and 26 weeks show only significant differences (denoted by asterisk * when comparing WT and Pyroxd1_N155S_ mice. **B)** Representative EM images of skin and aorta collagen fibrils from 3-month-old Pyroxd1_WT_ (WT) and Pyroxd1_N155S_ (S/S) mice. WT collagen fibrils appear less round and more densely packed compared to S/S collagen fibrils. **C)** Collagen fibril diameter measurements show no significant difference in mean fibre diameter distribution between WT and S/S mice, however there is a higher proportion of average-sized fibres in WT mice compared to S/S mice.

Supplementary Figure 11

**Supplementary Figure 11.** Additional Pyroxd1 KO cell proteomics. **A)** PCA plot shows appropriate clustering of the same genotypes. **B)** Volcano plots show significantly up- and down-regulated proteins are largely the same with or without thapsigargin treatment, with the exception of the tRNA ligase complex. **C-E)** GO clustering identifies protein changes in KO cells are mostly upregulation of components of the mitochondrial respiratory chain complex. **F)** KEGG pathway analysis identifies oxidative stress disorder pathways to be significantly upregulated in Pyroxd1 KO cells.

Supplementary Figure 12

**Supplementary Figure 12.** Additional Pyroxd1 KO skeletal muscle proteomics. **A)** PCA plot shows WT and KO replicates cluster appropriately. B) Volcano plot shows significantly up and down regulated proteins. **C-E)** GO enrichment analysis identifies mitochondrial pathways to be the most significantly down regulated in Pyroxd1 KO skeletal muscle.

Supplementary Figure 13

**Supplementary Figure 13.** Additional Pyroxd1_N155S_ skeletal muscle proteomics data. **A)** PCA plot shows SS samples cluster separately from all other samples. **B)** Volcano plots show no significant protein changes between samples from control mouse lines. **C)** Volcano plots between SS and SWT and GG samples show the same pattern of up and down regulated proteins as SS vs WT shown in Figure 8, with Bpnt2 and Phkb labelled here for clarity of labelling. **D)** GO comparisons for SS skeletal muscle vs all benign controls demonstrate the same pattern as seen for SS vs WT shown in Figure 8. **E)** KEGG pathway analysis reveals spliceosome as the most significantly changed biological process in SS skeletal muscle.
